# Immunoporosis: Role of Innate Immune Cells in Osteoporosis

**DOI:** 10.3389/fimmu.2021.687037

**Published:** 2021-08-05

**Authors:** Yogesh Saxena, Sanjeev Routh, Arunika Mukhopadhaya

**Affiliations:** Department of Biological Sciences, Indian Institute of Science Education and Research Mohali, Mohali, India

**Keywords:** immunoporosis, inflammation, innate immune cells, proinflammatory cytokines, ROS - reactive oxygen species, osteoporosis

## Abstract

Osteoporosis or porous bone disorder is the result of an imbalance in an otherwise highly balanced physiological process known as ‘bone remodeling’. The immune system is intricately involved in bone physiology as well as pathologies. Inflammatory diseases are often correlated with osteoporosis. Inflammatory mediators such as reactive oxygen species (ROS), and pro-inflammatory cytokines and chemokines directly or indirectly act on the bone cells and play a role in the pathogenesis of osteoporosis. Recently, Srivastava et al. (Srivastava RK, Dar HY, Mishra PK. Immunoporosis: Immunology of Osteoporosis-Role of T Cells. Frontiers in immunology. 2018;9:657) have coined the term “immunoporosis” to emphasize the role of immune cells in the pathology of osteoporosis. Accumulated pieces of evidence suggest both innate and adaptive immune cells contribute to osteoporosis. However, innate cells are the major effectors of inflammation. They sense various triggers to inflammation such as pathogen-associated molecular patterns (PAMPs), damage-associated molecular patterns (DAMPs), cellular stress, *etc.*, thus producing pro-inflammatory mediators that play a critical role in the pathogenesis of osteoporosis. In this review, we have discussed the role of the innate immune cells in great detail and divided these cells into different sections in a systemic manner. In the beginning, we talked about cells of the myeloid lineage, including macrophages, monocytes, and dendritic cells. This group of cells explicitly influences the skeletal system by the action of production of pro-inflammatory cytokines and can transdifferentiate into osteoclast. Other cells of the myeloid lineage, such as neutrophils, eosinophils, and mast cells, largely impact osteoporosis *via* the production of pro-inflammatory cytokines. Further, we talked about the cells of the lymphoid lineage, including natural killer cells and innate lymphoid cells, which share innate-like properties and play a role in osteoporosis. In addition to various innate immune cells, we also discussed the impact of classical pro-inflammatory cytokines on osteoporosis. We also highlighted the studies regarding the impact of physiological and metabolic changes in the body, which results in chronic inflammatory conditions such as ageing, ultimately triggering osteoporosis.

## Introduction

**Figure 1:**
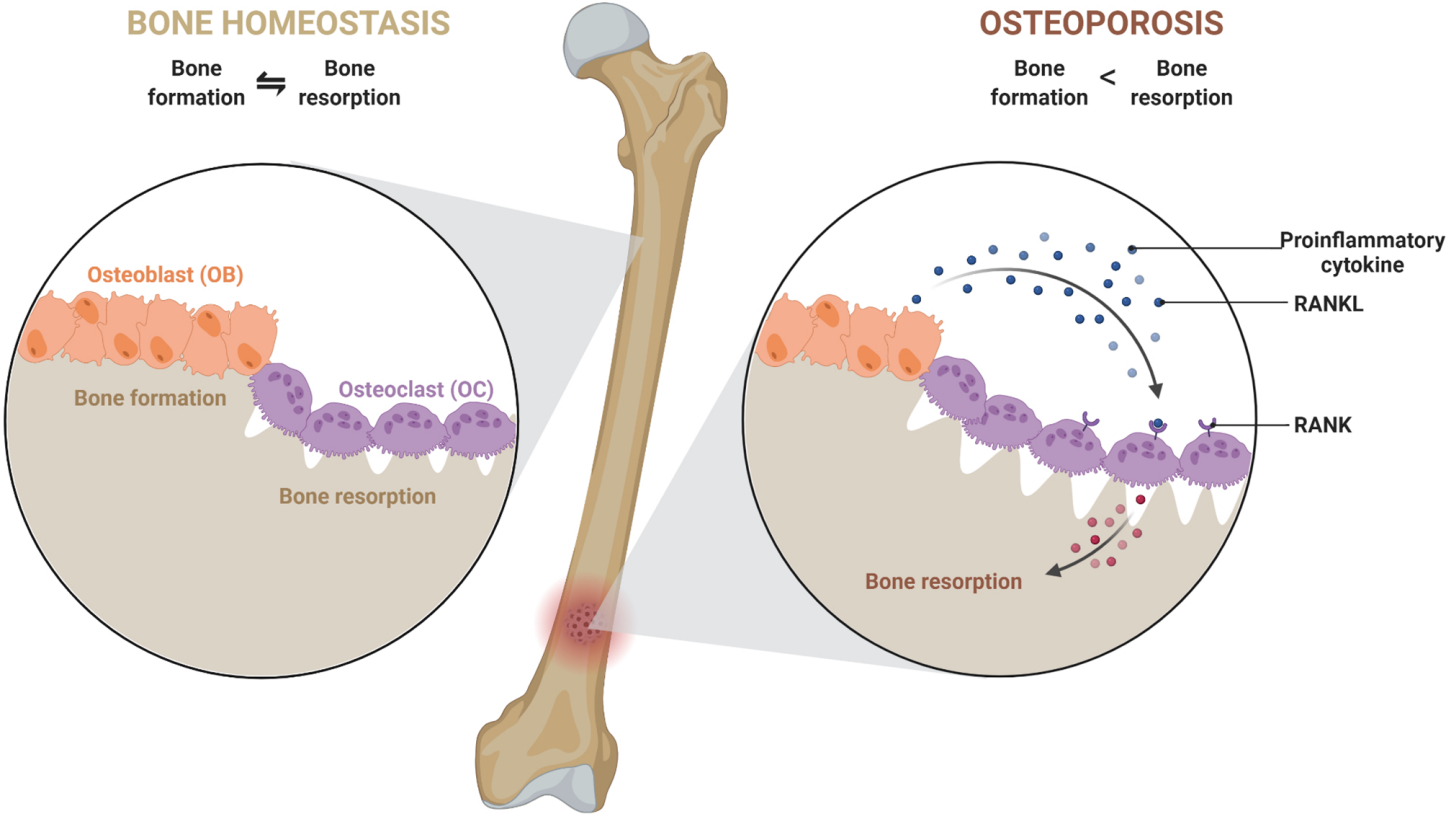
An imbalance in a dynamic equilibrium of bone remodeling leads to osteoporosis. Bone homeostasis is maintained by an equal amount of bone formation, and bone resorption carried out by osteoblast (OB) and osteoclast (OC). Under the influence of various mediators and cellular components, the equilibrium shifts towards greater bone resorption due to exacerbated osteoclast activity. This leads to a decrease in bone mineral density and causes osteoporosis.

A typical bone is composed of collagen, matrix proteins, calcium hydroxyapatite crystals, and cellular components. Different cellular components of a bone are osteoblasts (OBs), osteoclasts (OCs), osteocytes (OYs), stromal cells, mesenchymal stem cells (MSCs), hematopoietic stem cells (HSCs), *etc.* Among these, OBs and OCs play a major role in maintenance. OBs are of mesenchymal origin and have bone anabolic activity. They produce type-I collagen, matrix proteins (e.g., osteonectin and osteocalcin) to help calcium deposition in the form of calcium hydroxyapatite crystals. On the other hand, OCs, giant multinucleated cells of HSCs origin, demineralize the bone by releasing substances like hydrochloric acid and proteolytic enzymes, thus keep in check the anabolic activity of OBs (1). The antagonistic activity of OBs and OCs results in continuous formation and resorption of bone, a process called bone remodeling, which is necessary for maintaining calcium levels in the blood. Bone remodeling occurs in several specific spaces in the bone called bone remodeling compartments (BRC) (2). In a healthy bone, the bone homeostasis is regulated by sophisticated coordination among components of BRC’s through RANK (receptor activator of nuclear factor-κB), RANKL (ligand for a RANK receptor), and OPG (osteoprotegerin) interactions. OPG is a decoy receptor of RANKL. RANKL secreted by OBs interacts with RANK and triggers differentiation of precursor-osteoclast into bigger multinucleated active OCs. However, to keep bone resorption in check, OBs also secrete OPG, which competitively inhibits RANKL-RANK interaction (1–3). Any imbalance in the homeostasis can lead to bone anomalies such as osteopenia, osteoporosis, osteopetrosis, *etc.* In osteoporosis, there is an increase in the activity of osteoclast, leading to net bone loss (**Figure 1**).

Many different health conditions and medical procedures are correlated with osteoporosis, such as endocrine disorders (e.g., hyperparathyroidism, diabetes, premature menopause and low levels of testosterone and estrogen in men and women respectively, *etc.*), autoimmune disorders (e.g., rheumatoid arthritis or RA, lupus, multiple sclerosis, *etc.*), prostate cancer, thalassemia, liver dysfunction, organ transplant, *etc.* (4). Not only disease conditions, the later phase of life, aging, is also correlated with osteoporosis (1). During aging, epigenetic-metabolic changes in physiology drive chronic inflammation in the body resulting in osteoporosis (5). Hence, a diverse array of factors seems to be involved as the causative agents of osteoporosis. Though initially, it was thought that hormonal imbalance was the leading cause of osteoporosis, later in the 1970s, the role of the immune system first came to light (6, 7). Researchers observed that supernatant from the human PBMCs increased osteoclastic activity in fetal rat bone culture (6). In the past two decades, even more promising reports have emerged indicating firm involvement of immune cells in bone remodeling (8, 9). Age-driven changes in the status of immune cells explain the presence of chronic inflammation resulting in osteoporosis (10). The study of this intricate relationship between the immune system and skeletal system led to the establishment of a new field called “osteoimmunology” (11). Recently, Srivastava et al. have coined the term “immunoporosis” to emphasize the role of immune cells as a cause of osteoporosis (12). Another review by the same group has summarized the role of innate and adaptive immune cells in osteoporosis (13). In this review, we focus on the role of the innate immune cells in osteoporosis in a more detailed manner.

Cells of innate immunity are known to act immediately to various challenges to the body and cause ‘inflammation’, which has been observed as one of the major triggers of various bone disorders (13–15). According to a recent hypothesis published, inflammatory cell death, ‘pyroptosis’ of osteoblast, is critical in osteoporosis (16). Various signals that induce inflammation in the body include exogenous signals, such as PAMPs (Pathogen Associated Molecular Patterns) or endogenous signals, DAMPs (Death/Damage Associated Molecular Pattern), which abruptly challenge the immune system and results in acute inflammatory diseases. In addition, metabolic changes, tissue malfunctions or prolonged infections usually result in chronic inflammatory diseases. Therefore, inflammatory mediators produced in such cases play a key role in the co-morbidity of osteoporosis (17–20). ‘Focal infection theory’ is an old concept that assumes the foci of infection could cause systemic inflammatory diseases (as observed in periodontitis, psoriatic arthritis), resulting in osteoporosis (19, 20).

Innate immune cells are major producers of pro-inflammatory mediators. However, some of them share a common developmental niche with skeletal cells. Various reports suggest that the immune system is highly linked to the skeletal system and actively involved in the manifestation of the disease. In addition to the major producers of pro-inflammatory mediators, macrophages, monocytes, and DCs can act as precursors of osteoclasts (21, 22). Apart from macrophages, monocytes and DCs, other pro-inflammatory innate immune cells of myeloid origin, contribute to osteoporosis are neutrophils, eosinophils and mast cells (23–25). Innate cells of lymphoid lineage, such as NK cells and innate lymphoid cells (ILCs), also contribute to the manifestation of osteoporosis, majorly as producers of pro-inflammatory mediators (26, 27). Among the pro-inflammatory mediators that play a major role in osteoporosis, IL-6, TNF-α, IFN-γ, IL-1β, and ROS are worth mentioning. In this review, we will discuss the role and contribution of different types of innate immune cells and inflammatory mediators in osteoporosis (**Tables 1** and **2**).

**Table 1:** Function of different innate immune cell types and their role in osteoimmunology.

Cell type	Physiological role	Role in bone biology
**Macrophage**	Inflammation, phagocytosis, tissue repair	M1 macrophage promotes bone resorption *via* osteoclastogenesis (21, 28); M2 macrophage majorly promotes bone formation by stimulating differentiation of precursor cells into mature OBs (29, 30). However, in the absence of estrogen M2 macrophages can get differentiated in OCs (31). Osteal macrophages help in efficient bone mineralization (32).
**Monocyte**	Inflammation	Serves as a precursor to OCs, macrophages, and DCs (33). Helps in the recruitment of immune cells to the bone remodeling sites by producing chemokines (34).
**Dendritic cell**	Inflammation, antigen presentation	Can transdifferentiate to osteoclasts in the inflammatory milieu (35).
**Neutrophils**	Inflammation, phagocytosis	Promotes bone resorption by increased expression of mRANKL (36).
**Eosinophils**	Inflammation, allergic response	Found to be increased in number in vitamin D deficiency (37); Source of IL-31 and IL-31 found to be associated in postmenopausal osteoporosis (38)
**Mast cell**	Allergic response, inflammation	Triggers osteoclastogenesis by producing pro-inflammatory mediators such as, histamine, TNF-α & IL-6 (39, 40).
**NK cell**	Cellular cytotoxicity, ADCC, inflammation	Promotes osteoclastogenesis by producing RANKL & MCSF (26). Coculture with monocyte in the presence of IL-15 also promotes osteoclast formation (26).
**ILCs**	Tissue homeostasis, regulation of innate and adaptive immunity	Different subtypes of ILCs produce various factors like RANKL, GMCSF, IL-17 which are involved in multiple bone disorders (27, 41, 42).

**Table 2:** The role of classical pro-inflammatory cytokines in osteoimmunology.

Pro-inflammatory cytokines	Cellular sources	Role in bone biology
**IL-6**	OBs, OCs, Stromal cells, OYs, DCs, ILCs Macrophages, *etc.*	RANK-L mediated OCs activation, OCs transmigration (43, 44)
**TNF-α**	Osteoblast, T cells, B cells, macrophages, monocytes, NK cells, *etc.*	Increases RANK expression on the macrophage (45), increases RANKL production by the stromal cell (45), induces sclerostin in OYs (46), expand OCP pool (47), inhibiting differentiation, proliferation, and activities of osteoblast (48–50), degradation of osterix (48), inhibits differentiation of MSCs (50)
**IFN-*γ***	T cells, NK cells, B cells, ILC1, Neutrophils, Monocytes, Macrophages, MSCs, *etc.*	Fusion of OCs, T cell activation (51)
**IL-1β**	Osteoblast, T cells, B cells, Macrophages, *etc.*	OCs migration and activation (52–54) Plasminogen cathepsin-B and collagenase secretion (55), Downregulation ALP (56)

## Cells of the Myeloid Lineage

### Macrophages

Macrophages, one of the most potent inflammatory cells also act as the major sentinel cells. They are present in the tissues and can readily sense infection by various pathogens like bacteria, viruses, parasites, *etc.*, and provide a defense to the host system. They have the potential for phagocytosis as well as the induction of inflammatory responses. This ability comes from the presence of a broad range of pattern recognition receptors (PRRs) such as toll-like receptors (TLRs), nod-like receptors (NLRs), *etc.*, as well as scavenger receptors (SRs) (57). Similar sets of PRRs have been reported to modulate bone metabolism (58–61).

Macrophages are either tissue-resident or differentiated from blood monocytes in response to an inflammatory signal. The tissue-resident macrophages are present in different organs of the body and are known by different names, such as microglia in the brain and kupffer cells in the liver, *etc.* They are adapted uniquely to their location. The bone also possesses different kinds of macrophage populations: bone marrow macrophages (BMMs), OCs, and osteal macrophages or “osteomacs” (62). Osteal macrophages help in efficient osteoblast mineralization and bone formation (32). Depletion of osteal macrophages shows a decrease in bone mineral density (BMD) (32).

Different tissue microenvironment defines different phenotypes for tissue-resident as well as monocyte-derived macrophages. In addition to inflammation macrophage helps in tissue repair following injury and also maintains tissue homeostasis. To aid these activities (63), they show a great degree of plasticity and hence can undergo a transition between M1 and M2 phenotype depending on the microenvironment  (64). M1 is classically activated macrophages (inflammatory phenotype), and M2 is alternatively activated macrophages (reparative phenotype). Macrophage polarization drives bone remodeling activities. Pro-inflammatory cytokines such as, TNF-α and IL-6 can stimulate M1 polarization, whereas anti-inflammatory cytokines such as, IL-4 and IL-13 can stimulate M2 polarization (65), which are generally associated with bone catabolic and anabolic activities, respectively. However, an exciting study by Huang et al. reported that RANKL-induced M1 polarized macrophages display distinct properties compared to LPS and IFN-γ stimulated M1 macrophages (66). In a pathological scenario, it was observed that RANKL-induced M1 macrophages induce bone formation and help in increasing the osteogenic ability of MSC by increasing the expression of osteogenic genes such as *OPN, RUNX2*, *etc.*, while LPS and IFN-γ induced M1 macrophages shows bone destructive activity (66).

Numerous studies suggested a role of M2 macrophages in osteogenesis. Two groups have demonstrated that M2 polarized macrophages can stimulate MSCs, the precursor of OB cells, into mature OBs and increase bone mineralization *in vitro* (29, 30). Further, it has been observed that the co-culture of pre-osteoblastic cells with macrophage increased the osteogenic ability of pro-osteoblastic cells, and this attribute was enhanced by macrophage transition from M1 to M2 type (67). Based on this observation, it was suggested that a transient inflammatory phase is crucial for enhanced bone formation.

**Figure 2:**
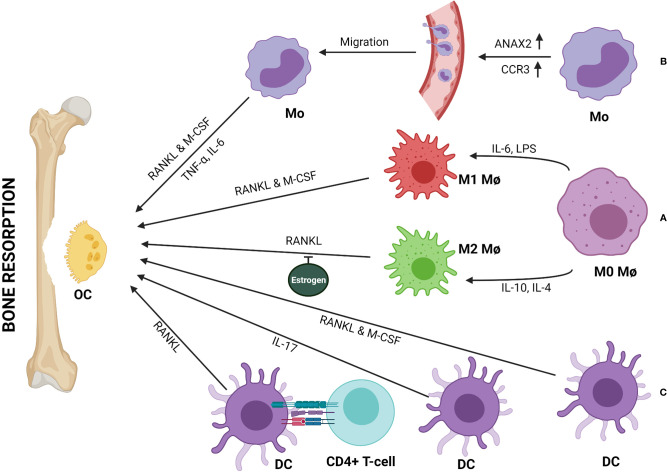
Monocytes (Mo), macrophages (Mφ), and dendritic cells (DCs) can be directly involved in bone resorption by differentiating into osteoclast (OC). **(A)** Both M1 and M2 subtypes of Mφ differentiate into OCs in presence of RANKL & M-CSF or RANKL respectively. Estrogen inhibits RANKL-mediated M2 differentiation to OCs. **(B)** Upregulation of ANAX2 & CCR3 help circulating Mo in trans-endothelial migration and recruitment to the bone remodeling site, where Mo to OC differentiation happens either in the presence of RANKL & M-CSF or TNF-α and IL-6. **(C)** DCs can transdifferentiate into OCs in presence of RANKL & M-CSF or IL-17. Further, immune interaction between CD4+ T-cells and DCs can form OCs in a RANKL/RANK-dependent manner.

M1 macrophage serves as a precursor of osteoclast (28). Researchers had observed that the osteo-inductive mediators, such as bone morphogenetic protein (BMP) 2 and 6, were reduced when macrophages were stimulated by a known M1-phenotype inducer (68). M1 inducer, such as LPS induces a massive production of pro-inflammatory cytokines and triggers osteoclastogenesis in RANKL-dependent or -independent manner leading to bone destruction (**Figure 2A**) (21). Multinucleation of macrophages is driven by RANKL-dependent or -independent signaling pathways that bring about the changes essential for multinucleated osteoclast differentiation and formation (68–70). One of the necessary and key changes observed in macrophage to osteoclast differentiation is the changes in energy metabolism. A report using RAW 264.7 murine macrophage cell line and bone marrow-derived macrophages (BMDMs) suggested that lysine promotes M1 & M2 activation, whereas tyrosine and phenylalanine have opposite effects (71). Another report indicated that differentiated osteoclasts are rich in lysine degrading proteins and show enhanced biosynthesis of tyrosine and phenylalanine (72). These two reports suggested that inhibition of polarization of macrophage enhances osteoclast differentiation. Additionally, there is an increase in mitochondrial biogenesis in RANKL-induced osteoclastogenesis (73). Consequently, the increase in oxidative phosphorylation allows increasing bone resorption by osteoclasts. In another report, it is observed that there is an increase in GLUT1 and other glycolytic enzymes during osteoclast differentiation (73). Both glycolysis and oxidative phosphorylation thus play an important role in osteoclastogenesis. Recent evidence suggested that glucose transporter expression depends on RANKL (74). It explains why macrophage to osteoclast differentiation and bone resorption is associated with an increase in energy metabolism (75).

A study suggested that, M1/M2 macrophage ratio increases in the bone-marrow of ovariectomized (OVX) osteoporotic mice. In the absence of estrogen, M2 macrophages differentiate into osteoclast upon stimulation with RANKL (**Figure 2A**) (31). Thus, estrogen protects M2 macrophages from RANKL stimulation. Therefore, M1/M2 ratio and estrogen are related to the pathogenesis of postmenopausal osteoporosis.

It is well accepted that macrophages play an essential role in the pathogenesis of inflammatory disease rheumatoid arthritis (RA) by producing pro-inflammatory cytokines like TNF-α, IL-1β, IL-6 that can drive osteoclastogenesis and bone destruction. Similar contributions by macrophages were also observed in osteoarthritis (OA) and peri-implant osteolysis (76). The role of macrophages in RA and OA has been elaborately discussed in other reviews (77, 78).

Inhibitor studies have helped to elucidate the signaling pathways involved in the RANKL-mediated osteoclastogenesis. Researchers have observed that a Chinese herb, Bergapten inhibits RANKL-induced osteoclastogenesis by suppressing the degradation of IκBα (inhibitor of NF-κB) (79). IκBα keep NF-κB in the cytoplasm by binding to it; degradation of IκB is necessary for translocation of NF-κB to the nucleus and perform its functions. Researchers have also observed that Bergapten attenuates JNK phosphorylation (79). Icariin (ICA) inhibits RANKL-induced osteoclast formation by downregulating signaling mediator TRAF6 (adaptor molecule associated with RANK complex) and further affecting the NF-κB pathway (80). Additionally, it was also observed that ICA inhibits ERK phosphorylation which subsequently leads to a decrease in NFATc expression, which is also a crucial transcription factor for osteoclastogenesis (80). Sappanone A was shown to inhibit RANKL-induced osteoclastogenesis by inhibiting the phosphorylation of AKT and subsequently suppressing the activation of NFATc1 and other osteoclastogenic markers (81). It is interesting to note that RANKL stimulation induces activation of all the three major MAPKs (ERK, JNK, p38); however, only the p38 signaling pathway plays a crucial role in RANKL-mediated differentiation of macrophage to OCs (82). So far no natural inhibitors have been found to inhibit p38 signaling pathway in osteoclastogenesis.

(82, 84)With aging macrophages show an array of dysfunction, including defect in autophagy, morphological changes, and dysregulation of pro-inflammatory cytokine production resulting in age-related altered immune function (10, 83). A study shows significant increase in M1-polarised macrophages in aged mice (84). Aged macrophage shows amplified production of inflammatory mediators (85, 86). Therefore, in macrophage from older people displays an activated phenotype and increased basal level inflammation (86). It is also reported that macrophage polarization dysfunction is related to impaired bone healing in aged mice and rat populations (86–88). Altogether, macrophage dysregulation contributes to a chronic low-grade inflammation during aging, called Inflammaging, which often correlates with osteoporosis.

Apart from inflammatory responses, recent studies have recognized new regulatory mechanisms of macrophages in osteoporosis. A microarray study on RANKL and CSF1 treated *vs.* non-treated BMMs identified differentially expressed circular RNAs (circRNAs) and found that circRNA_28313 was significantly induced in treated cells. Further, it was observed that knockdown of circRNA_28313 significantly inhibited macrophage differentiation to OCs *in vitro* and OVX-induced bone resorption *in vivo* in mice. Bioinformatic analyses revealed that mir-195a microRNA interacts with 3’UTR of CSF1 in non-treated cells (89). However, circRNA_28313 relieves miR-195a-mediated suppression on CSF1 *via* acting as a competing endogenous RNA (ceRNA), modulating the osteoclast differentiation in BMM cells (89). Another miRNA, miR-128, regulates osteoclastogenesis of BMMs through miR-128/SIRT1/NF-kB signaling axis (90). The overexpression or inhibition of miR-128 can increase or decrease macrophage-derived OCs (90). Further reports suggested that miR-506-3p can selectively inhibit NFATc1 in RANKL-induced activated BMMs in rats and minimize the release of bone resorption enzymes (91). The heterogeneity and plasticity of macrophages make them a critical player in bone homeostasis. A more in-depth study is required to understand the role of macrophages in immunoporosis. Modulation of macrophage phenotype could be a potential therapeutic target in dealing with osteoporosis.

### Monocytes

Monocytes constitute 10% of total leukocytes in humans and 2-4% in mice (92). The precursors of monocyte arise from HSC in the bone marrow and finally undergo subsequent differentiation to become a committed monocyte progenitor (cMoP) (92). Similar to macrophages, monocytes also exist as different subsets exhibiting different phenotypes and functions. Different subsets of monocyte show distinct functions during steady-state and inflammation. The inflammatory monocytes show high levels of C-C chemokine receptor 2 (CCR2) and low levels of CXC3 chemokine receptor 1 (CX3CR1), whereas the patrolling monocytes show the reverse expression (93). Their recruitment to the inflammatory site is predominantly CCR2 dependent (94). Traditionally, it is considered that monocyte extravasate from blood vessels to the site of inflammation and differentiates into macrophages or dendritic cells, and contributes to the inflammatory process and repair (95). However, a study demonstrated that CCR2-expressing pro-inflammatory monocyte transitioned into CX3CR1-expressing reparative monocyte (96). However, Jakubzick et al., in their study, reported that monocytes can retain their markers or their identity without differentiating into macrophages and DCs while moving through tissues (97). These studies suggest that the monocyte can participate in the inflammatory process directly apart from acting as precursors only. Accordingly, circulating monocyte plays some crucial role by serving as osteoclast precursor and participating in bone remodeling by producing cytokines (33, 34). Recent reports indicate that erythromyeloid progenitor-derived monocytes (EMP-monocyte) also contribute to this pool of circulating monocytes apart from major contributor HSCs-monocytes (98–100). Interestingly, EMP-monocytes, which reside in the adult spleen postnatally, transmigrate to the bone marrow where they differentiate into functional OCs along with HSCs derived OCs and helps in bone repair in fracture scenarios (98). Similar to macrophages, monocytes also undergo metabolic changes like, increase in glucose uptake, oxidative phosphorylation etc. during differentiating into osteoclast (101). Different environmental cues drive different metabolic changes and as a result, monocyte responds differently. The three phenotypic forms of circulating monocyte in human peripheral blood are Classical (CD14^++^ CD16^-^), intermediate (CD14^++^ CD16^+^), and non-classical (CD14^+^CD16^++^), which differentiate into osteoclast with different order of resorbing ability, that is, normal, high, and non-absorbing, respectively (75). Reports suggest that non classical human monocyte expresses respiratory chain metabolism whereas classical monocyte depends on carbohydrate metabolism and primed more towards anaerobic energy production (102).

In an infection scenario, intermediate monocytes take the lead to become high bone absorbing osteoclast and may result in bone weakening (74), indicating monocytes could also play a critical role in bone disorders (75). Researchers have made some observations towards it. The Association of monocyte with post-menopausal osteoporosis in Caucasian women was shown by Zhang et al. (29, 102, 103). Network-based proteomics analysis of peripheral blood monocytes (PBM) showed significant downregulation of proteins encoded by four genes, namely*, LOC654188, PPIA, TAGLN2, YWHAB* whereas, upregulation of proteins encoded by three genes, namely *LMNB1, ANXA2P2, ANXA2*, in extremely low- *versus* high-BMD subjects (103). Proteomics analysis of PBM of low-BMD subjects showed upregulation of the ANXA2 protein (104). Cellular studies revealed that ANXA2 is important in monocyte migration across the endothelial barrier. Thus, the elevation of ANXA2 probably stimulates the higher migration rate of monocyte from blood to the bone tissue and then differentiate to OCs and contribute to bone-resorbing activity (**Figure 2B**) (104).

Additionally, a microarray study on circulating monocytes in humans suggested that three genes, *CCR3* (C-C chemokine receptor type 3), *HDC* (histidine decarboxylase, a histamine synthesis enzyme), and *GCR* (glucocorticoid receptor), might contribute to bone metabolism and homeostasis. These three genes are found to be upregulated in subjects with low BMD (105). These genes mediate monocyte chemotaxis, which can lead to monocyte infiltration in bone tissue, histamine production, which induces local inflammation and can mediate OC formation, and glucocorticoid sensitivity which promotes OC formation (105). *In vivo* gene expression profiling in human monocyte suggested upregulation of STAT1 and IFN pathway genes in low BMD groups (106). Based on additional observations, the researchers proposed that probably in peripheral blood, IFN-mediated by *STAT1* stimulates circulating monocytes to produce cytokines (such as IL-1, TNF, CXCL10, and IL-15) that increase the bone resorption function of osteoclast. Daswani et al. provided further insight into monocyte proteomics, which revealed the involvement of phosphorylated heat-shock protein 27 (HSP27) in low BMD subjects (107). They have observed elevation of total phosphorylated HSP27 (pHSP27) in monocyte of low BMD subjects and validated that pHSP27, not a chemoattractant itself but acts as an actin reorganizer, facilitating migration (107). As Hsp27 inhibits stress-induced apoptosis, and since osteoclast formation involves ROS generation, the anti-apoptotic activity of pHSP27 may foster monocyte survival and hence more precursor for osteoclastogenesis (107). Transcriptome study identified the downregulation of two apoptosis-inducing genes, death-associated protein 6 (DAXX) and polo-like kinase 3 (PLK3), in low BMD subjects (108). This report supported the fact that due to monocyte survival, more precursors are available to augment osteoclastogenesis and hence osteoporosis.

It was observed that the *SBDS* gene, which is responsible for the disease SDS (Shwachman-Diamond Syndrome showing skeletal defects), plays a role in monocyte migration and fusion before osteoclastogenesis (109). Sbds mutant showed a decrease in Rac2 (GTPase required in cytoskeletal remodeling for migration) and RANKL-mediated DC-STAMP (required for fusion of osteoclast precursor) levels. This fusion defect reduces osteoclastogenesis. Reduced osteoclastogenesis expects osteopetrosis phenotype. Surprisingly, SDS patients show an osteoporotic phenotype. The potential explanation for this phenomenon is that since a reduced number of TRAP-positive multinucleated OCs are still present, there is no complete uncoupling of bone remodeling homeostasis, which probably triggers a shift of MSCs towards adipocyte cell lineage instead of osteoblast. Other observations supporting this phenomenon have been reported earlier by the same group (110).

### Dendritic Cells

Dendritic cells are majorly antigen-presenting cells (APCs) endowed with abilities to activate the adaptive immune response. They express high levels of MHC class II and co-stimulatory molecules such as CD80 and CD86 which are required for antigen presentation. DCs are distributed throughout the body and survey for external and internal dangers using a broad range of PRRs such as TLRs, CLRs, NLRs, *etc.* Dendritic cells can be divided into three subgroups: plasmacytoid DCs (pDCs) derived from lymphoid progenitors, classical or conventional DCs (cDCs) derived from both lymphoid or myeloid progenitors, and monocyte-derived DCs (moDCs). pDCs function against viral infections by secreting type I IFNs (111). In addition to pDCs, cDCs and moDCs play a role in providing defense against other microbes.

However, the profound effect of DCs on bone metabolism has been widely recognized recently. DCs contribute to inflammation-mediated osteoclastogenesis and take part in inflammatory bone disease. Using an *in vivo* model, Maitra et al. reported the osteolytic potential of DCs (112). They observed that dendritic cells recruit to bone inflammatory sites and participate in bone resorption (112). In addition, DCs can activate T-cells by acting as APCs, and the activated T-cells produce cytokines and soluble factors that drive bone remodeling (113). It was also observed that DCs directly interact with T-cells to form aggregates which play a role in bone pathologies like synovitis and periodontitis (114, 115). In a recent study, the role of DCs in manifesting osteoporosis in OVX mice was reported (116). Estrogen is known to regulate the number of DCs that express IL-7 and IL-15. In the absence of estrogen, the DCs sustains for long and express more IL-7 and IL-15, which, together, induces a subset of memory T-cell to produce IL-17A and TNF-α in an antigen-independent manner. The cytokines so produced drive inflammation-mediated bone loss (116). There are also reports suggesting a more direct role of DCs in osteoclastogenesis. It has been observed that the DCs can potentially trans-differentiate and fuse to form OCs, and this fusion is faster and efficient than monocyte fusion. There is downregulation in the expression of 3997 genes for differentiation from monocytes to OCs, while there is upregulation in the expression of 3821 genes. However, when immature dendritic cells differentiate into OCs, there is downregulation of only 2107 genes and upregulation of 1966 genes suggesting that DCs are more closely related to osteoclast than monocytes (117). The newly formed OCs can summon more DCs by inducing chemotaxis of DCs, and the OC-DC loop continues to increase bone destruction (117).

Studies showed that DCs can trans-differentiate to OCs in the presence of RANKL and macrophage colony-stimulating factor (M-CSF) (**Figure 2C**) (35, 118). Another study suggested that activated DCs (bone-marrow-derived and splenic CD11c^+^ cells) upon interaction with T helper-cells (CD4^+^ T cells or Th) can develop into functional OCs (TRAP^+^CT-R^+^cathepsin-k^+^) in RANKL/RANK-dependent manner (**Figure 2C**) (119). In a report, Th17 cells were shown to play a role in the trans-differentiation of DC to OCs (120). It has been observed in RA patients that inflammatory milieu can recruit Th17 cells, which produce a huge amount of IL-17 to stimulate RANKL production by bone stromal cells and promotes nuclear fusion of immature DCs *via* IL-17R (IL-17 receptor) (**Figure 2C**) (117). T-cells not only augment trans-differentiation of DCs to OCs but also can inhibit it by producing cytokines like IFN-γ. T-cell-derived IFN-γ can inhibit RANKL signaling by blocking TRAF6 to inhibit osteoclast maturation and activation (8). Hence, T-cells could act as a balance switch in mediating DC-OC trans-differentiation. Moreover, in specific conditions, DCs are known to produce TGF-β which is a potent anti-osteoporotic molecule (121–123). This indicates a potential alternative role of DCs in osteoporosis. A more in-depth study is required to understand the full potential of DCs in the induction of immunoporosis.

### Neutrophils

Neutrophils are the polymorphonuclear (PMN) phagocytic cells and the first leukocyte to be recruited at the site of inflammation (124). They make up 40-60% of leukocytes in the human blood. Neutrophils contain different granules, which are a source of several anti-microbial molecules. They continuously monitor for microbial infections and kill the pathogen by various mechanisms that include phagocytosis, production of ROS, and molecules like granzyme-B and perforins (125, 126). In addition, neutrophils also exhibit a unique strategy of immobilizing and killing microorganisms by extruding a meshwork of chromatin fibers covered with granule-derived antimicrobial peptides and enzymes. These are called neutrophils extracellular traps (NET), and the mode of killing is termed as NETosis (127).

Current research has emphasized the diverse role of neutrophils beyond killing pathogens. Neutrophils respond to different signals in the inflammatory milieu by producing cytokines and chemokines, which can regulate inflammation and other pro-inflammatory cells (128, 129). In contradiction to the old belief as short-lived innate effector cells, recent evidence suggested the role of neutrophils in regulating adaptive immune response (130). Thus, neutrophils interact with both innate and adaptive arms of the immune system and differentially respond depending on the context.

**Figure 3:**
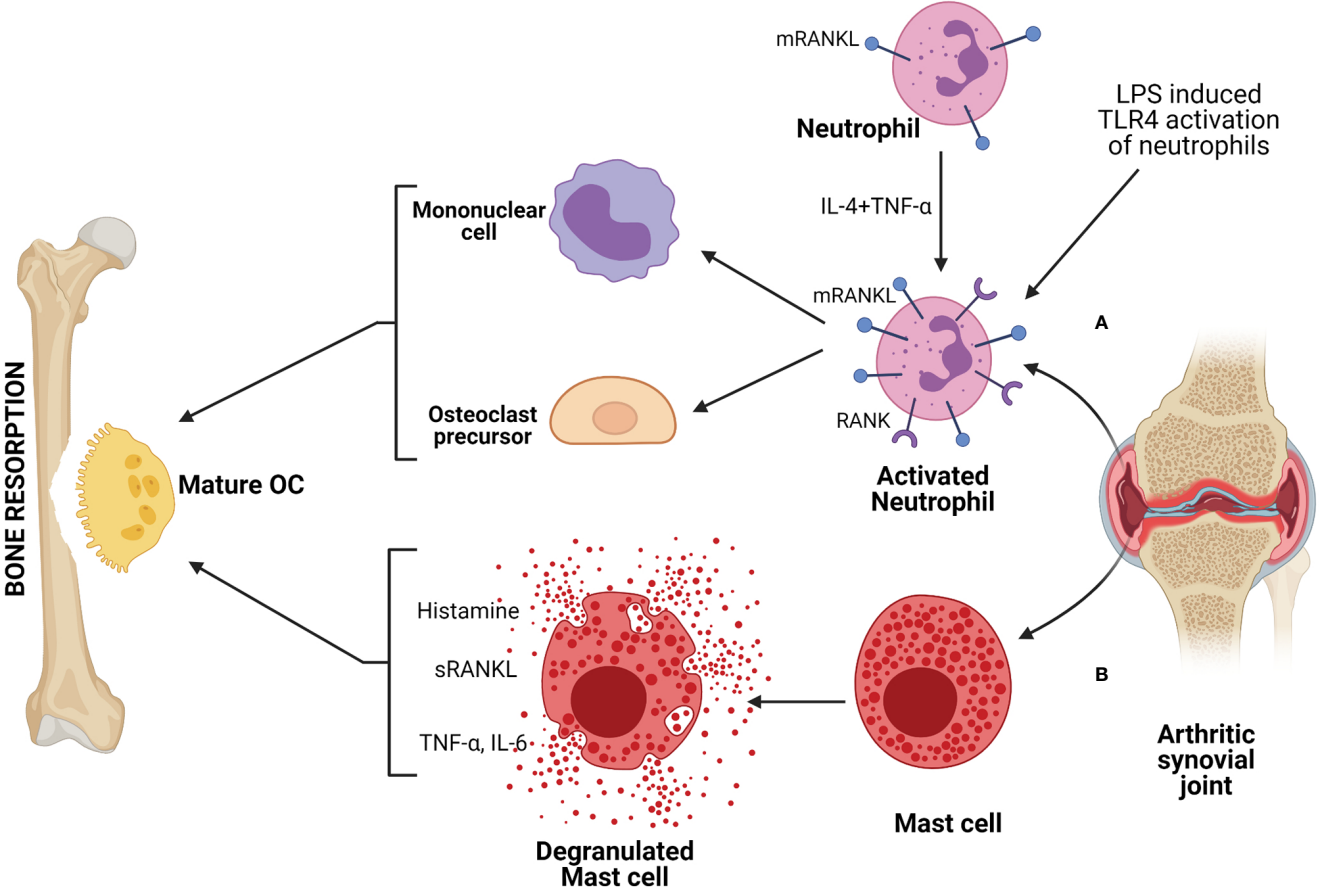
Neutrophils and mast cells are involved in bone resorption by producing factors that can drive osteoclastogenesis. **(A)** Activated neutrophils, either stimulated with TLR agonist or derived from patients with inflammatory disease, such as, from SF (synovial fluid) of RA (rheumatoid arthritis) patient, express higher membrane bound RANKL (mRANKL) and act on OC precursors or mononuclear cells to form mature and functional OCs, resulting in bone resorption. **(B)** Mast cells derived from SF of arthritic patient produces mediators like histamine, TNF-α, IL-6 that drive osteoclastogenesis and further bone resorption.

Neutrophils are also involved in the pathophysiology of various diseases, including inflammation-mediated bone loss (131). Moreover, neutrophils can produce chemokines and recruit pro-osteoporotic cells such as Th17 (12, 131). A strong correlation was indicated between an increase in RANKL positive neutrophils with inflammatory disease conditions and a decrease in BMD (23). A report demonstrated that neutrophils from the blood of a healthy individual express membrane-associated RANKL (mRANKL) while RANK expression depends on IL-4 and TNF-α stimulation (**Figure 3A**) (132). Interestingly, it was observed that synovial fluid (SF) neutrophils from RA patients express both mRANKL and RANK and also secrete OPG (131, 132). This observation that inflammatory neutrophils impetuously express RANK whereas healthy blood neutrophils express only after stimulation gives an insight into the involvement of neutrophils in bone remodeling. The evidence of inflammatory neutrophils expressing RANK could be related partly to acquiring a dendritic cell phenotype and further activating T-cells in RA condition (133). A study reported that mRANKL of TLR4-activated neutrophils induce osteoclastic bone resorption (**Figure 3A**) (36). The mRANKL of activated neutrophils act on both OCs and their mononuclear precursors, converting them into mature and functional OCs that contribute to bone resorption. Interestingly, they have demonstrated that the membrane fraction of activated neutrophils can augment the osteoclastogenic effect but not the culture supernatant, suggesting the importance of the involvement of mRANKL (22, 36). Another study reported that there is an increase in RANKL positive neutrophil in the blood of chronic obstructive pulmonary disease (COPD) patients compared to smokers and healthy controls, and it is related to low BMD (24).

Neutrophils can augment bone loss; however, some reports suggested that neutrophils play a to reduce bone loss by maintaining a homeostatic condition. The defective neutrophil recruitment in leukocyte adhesion deficiency type I (LAD-I) disorder results in IL-17 driven inflammatory bone loss (134). There is a defect in the expression or function of β2 integrin or related adhesion molecule in LAD-I disorder. Due to this defect, there is an impairment in neutrophil extravasation to the inflammatory site. The absence of neutrophils results in unrestrained production of IL-23 from macrophages, which in turn triggers IL-17 production from T-cells and can drive IL-17-mediated inflammatory bone loss. Another report supported the role of neutrophils in preventing inflammation-mediated bone loss. Gif1 is a molecule in HSC development, and a defect in *Gif1* causes severe neutropenia (135). This condition can induce osteoporosis depending on pathogen load and systemic inflammation (135). Hence, neutrophils can be a very critical player in the regulation and manifestation of osteoporosis.

### Eosinophils

Eosinophils are known to be involved in the pathogenesis of various allergic and inflammatory diseases (136). However, recently reported association of Vitamin D (VD) deficiency with an increased number of blood eosinophils indicates potential role of eosinophils in bone biology. Moreover, VD, which is a well-known osteoprotective molecule, decreases production of IgE as well as, release of peroxidase from eosinophil and while increases the production of the osteoprotective cytokine IL-10 (37, 137, 138). Eosinophils play a role in the manifestation of various inflammatory diseases (138), including chronic obstructive pulmonary disease (COPD), eosinophilic esophagitis (EoE), *etc.* (139, 140). Literature indicates a strong correlation between COPD and osteoporosis (141). However, the reason identified for the co-morbidity of osteoporosis is the use of a steroid-based treatment regime. Steroid-based treatments are frequently used to manage symptoms in COPD and EoE. Although, the role of eosinophils with steroid-induced osteoporosis is under scrutiny and not fully understood yet (141, 142).

Eosinophils carry out allergic responses by producing inflammatory mediators such as ROS, cysteinyl leukotrienes, and various cytokines and chemokines (143). Transcription factors such as NF-κB mounts such allergic inflammatory responses. However, such transcription factors can also induce osteoclastogenesis in an inflammatory condition. Interestingly, eosinophils are the source of IL-31 in an inflammatory skin condition called Bullous pemphigoid (BP) (144). IL-31 is a pro-inflammatory cytokine that serves as a biomarker for allergic disease. It is involved in the regulation of cell proliferation and tissue remodeling (145). It is reported to be involved in the regulation of the transcription factors and cytokines that are associated with osteoporosis. It is also observed that there is an increase in serum IL-31 level in post-menopausal women with a decrease in BMD, correlating with age (38). Association of eosinophil with IL-31 suggests that eosinophil may play a role in the manifestation of osteoporosis in an exacerbated allergic and autoimmune inflammatory disease condition. However, more studies are required to understand the contribution of eosinophil towards osteoporosis.

### Mast Cells

Mast cells are the tissue-resident immune cells that originated from pluripotent progenitor cells of bone marrow. Mast cell progenitors migrate into the tissue, where they differentiate and mature (146). Though they are best known for fostering allergic responses, they are also involved in numerous physiological functions and pathophysiology of various diseases (147). Mast cells are the first cells to respond to invading foreign entities as they are present at the tissue boundaries. They can be activated by PAMPs *via* PRRs such as TLRs or complement systems. Mast cells store a wide variety of preformed inflammatory mediators, including histamine, TNF-α, IL-6 as well as proteases in their secretory granules (148). As these inflammatory mediators are known to regulate bone homeostasis and involved in pathogenesis of various bone disorders, mast cells could be a probable candidate associated with bone disorder. Indeed, few reports suggested that there was increase in number of mast cells in the patient with reduced bone density and associated with post-menopausal osteoporosis (149, 150). Experimental evidences suggested that in OVX-induced estrogen depletion there was an increase in numbers of mast cells as well as osteoclasts (151, 152). These observations indicated that mast cells probably promote osteoclast formation in estrogen-deficient conditions. Treatment with calcium and promethazine (a blocker of the histamine H1 receptor) to post-menopausal women helped increase BMD in comparison to calcium alone. The observation that H1 receptor blocking resulted in the termination of osteoclast formation by mast cell supernatant suggested that the histamine, one of the main preformed mediators of mast cell, has a role in the reduction of BMD (153). Other reports indicated that estrogen affects mast cells and the release of its mediators, suggesting estrogen has an inhibitory effect on the osteoclast-inducing potential of mast cells (148). However, there are contradicting reports which indicated that estrogen could induce degranulation of mast cells since estrogen did not stimulate degranulation in ERα (estrogen receptor) knockout mice (154).

Mast cells have been suggested to be involved in the RA disease condition. Similar to other immune cells, mast cells are also abundantly found in inflamed synovial joints of RA patients. Mast cell mediators such as histamine and tryptase were found to be increased in SF (39, 40). Mast cells also contribute to the inflammatory milieu of SF as activated mast cells can produce mediators like TNF-α, IL-6, etc., which have the ability to induce osteoclastogenesis (**Figure 3B**). Even increased levels of RANKL found in the synovial tissue of RA patients could be contributed by mast cells as activated mast cells secrete RANKL (155). In a mice model of CIA (collagen-induced arthritis), reduction in T-cell numbers (both CD4^+^ and CD8^+^) along with reduced IFN-γ and IL-17 were observed upon depletion of mast cells (156). This indicated that mast cells might be involved in regulating T-cell expansion and Th1 and Th17 polarization, which is further involved in T-cell-driven arthritis. Various reports suggested that mast cells also play a role in OA. This could be due to mast cell-driven increased pro-inflammatory responses. An increase in the number of mast cells, as well as histamine or tryptase levels, were observed in SF of OA patients (157–159). It has been observed that in OA, mast cells are activated *via* IgE/FcϵRI receptor axis (158). Another report showed that synovial mast cells from OA patients produce TNF-α upon stimulation *via* high-affinity receptor of IgG (160). The link between mast cells and bone was also exemplified by the presence of mast cells during fracture healing. A gradual increase in mast cell numbers was observed in periosteal fracture callus, followed by a decrease during callus remodeling (161). The highest number of mast cells was also found in the vicinity of OCs and bone resorption sites during callus remodeling, indicating that mast cells could be involved in regulating osteoclast activity (162). Further investigation is required to understand the mechanism of action of mast cells in the physiology of bone turnover and bone disorders.

## Cells of the Lymphoid Lineage

### NK Cells

Natural killer (NK) cells are developed from HSCs in the bone marrow. However, recent evidence suggested that they can also develop and mature in secondary lymphoid tissues (SLTs) and show some adaptive features such as memory generation (163). In the 1970s, NK cells were described as large granular lymphocytes with the ability of “natural cytotoxicity” against various tumor cells. In recent times, it is now appreciated that apart from cytotoxicity against tumor cells, it is also capable of showing cytotoxicity against virus-infected and stressed cells (164, 165). NK cell surveillance system consists of various cell surface activating and inhibitory receptors that help identify and kill target cells (166). Additionally, they can perform antibody-mediated cell cytotoxicity (ADCC), making it a potent effector cell of the humoral response. They also exhibit cytokine-producing effector function. Upon engagement with target cells, they can secrete various pro-inflammatory cytokines and chemokines and, thus, regulate other immune cells’ functionality by modulating the local milieu (167). NK cells also play a crucial role in maintaining homeostasis and immunoregulation *via* control of T-cells activity.

**Figure 4:**
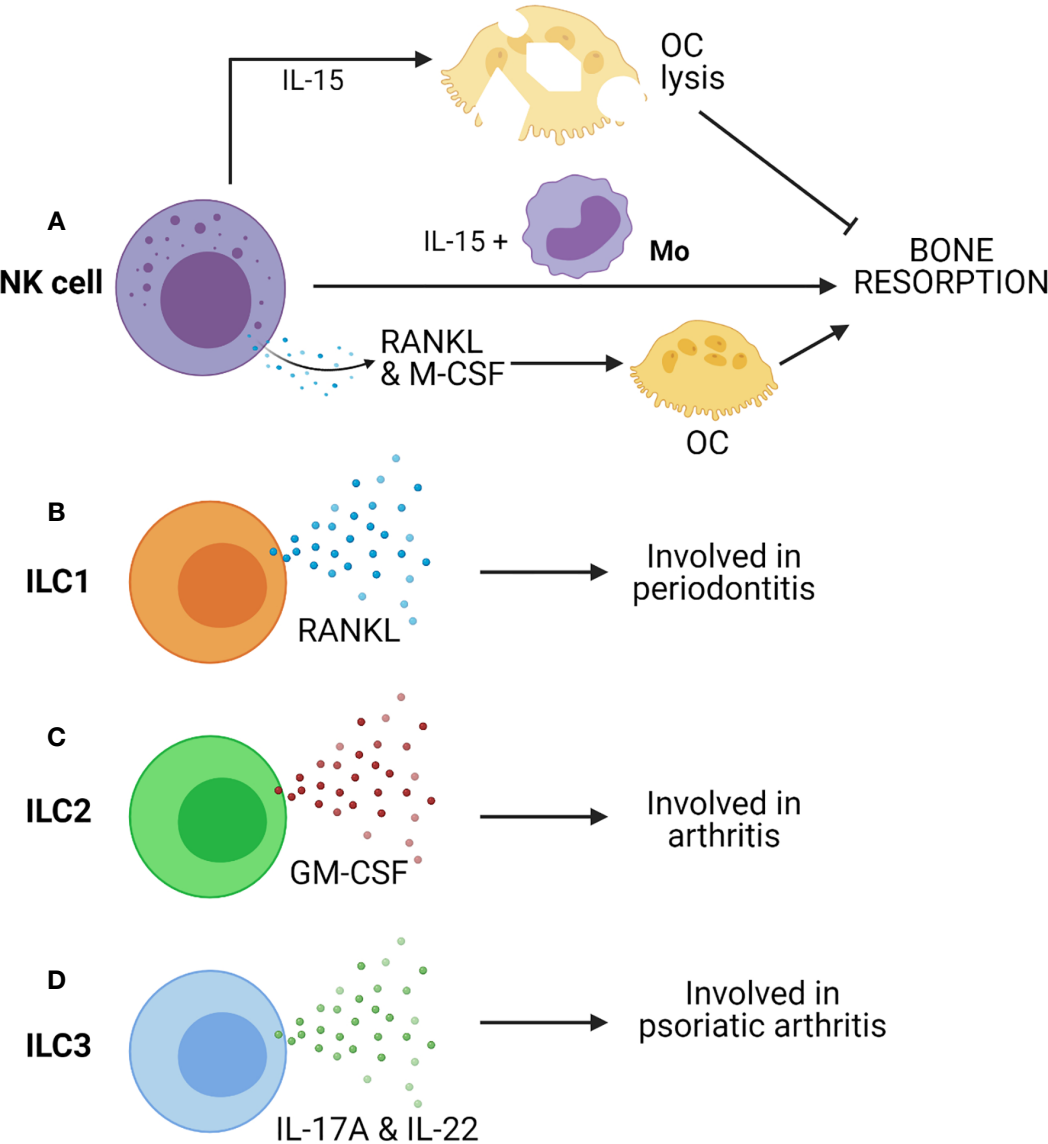
Innate cells from lymphoid lineages, such as Natural killer (NK) cells and Innate lymphoid cells (ILCs) can contribute to bone resorption. **(A)** IL-15 activated NK cells can induce OC lysis and inhibits bone resorption. However, co-culture of NK cells with monocytes (Mo) triggers bone resorption in presence of IL-15. Further, NK cell-mediated production of RANKL & M-CSF can drive osteoclastogenesis. **(B)** ILC1 produces RANKL and is associated with periodontitis. **(C)** ILC2 produces GM-CSF and is associated with arthritis. **(D)** ILC3 produces IL-17A & IL-22 and is associated with psoriatic arthritis.

Since NK cells are well poised to carry out inflammatory processes and cytotoxicity, they are involved in the manifestation of inflammatory diseases. There are reports which displayed the presence of NK cells in inflamed synovial tissues at an early stage of RA (168, 169). Such NK cells express M-CSF and RANKL, potent activators of osteoclastogenesis (**Figure 4A**). These molecules are further upregulated by IL-15, which is abundantly present in the synovium of RA patients (170). Soderstrom et al. showed that NK cells from SF of RA patients trigger efficient formation OCs from monocyte (**Figure 4A**) (26). They had also demonstrated that OCs formed from monocyte when co-cultured with NK cells were capable of eroding bone in the presence of IL-15 but not in the absence of it (26). In the CIA mice model, many synovial NK cells express RANKL suggesting the role of NK cells in bone erosion, and it was observed that depletion of NK cells prevents bone erosion in CIA.

A study suggested that IL-15 activated NK cells can kill OCs (**Figure 4A**). In the presence of IL-15, it seems that NK cells’ action is contradictory to the report mentioned above (171). IL-15 upregulate leucocyte function-associated antigen-1 (LFA-1) and DNAX accessory molecule-1 (DNAM-1) on NK cells. These are ligands of ICAM-1 and CD155 (PVR) receptors, respectively. These receptors are present on OCs, and they are essential for their development, function, and interaction with stromal cells (172). Receptor blocking studies between OCs and NK cells has displayed restoration of bone resorption. Thus, IL-15 activated NK cells kill OC *via* LFA-1 and DNAM-1 (171). Therefore, NK cell-mediated inhibition of osteoclast is contact-dependent, although IL-15-activated NK cells produce soluble factors like IFN-γ that can be anti-osteoclastogenic. Thus, NK cells can control or augment osteoporosis depending on the tissue microenvironment. More in-depth study is required to understand the NK cell-mediated regulation of bone remodeling and osteoporosis.

### ILCs

Innate lymphoid-like cells or ILCs are the heterogeneous populations of cells that arise from the lymphoid lineage (173). Although they lack antigen-specific receptors, upon tissue damage or pathogen invasion, they can sense changes in the local milieu by cytokine receptors and modulate subsequent antigen-specific lymphocyte responses. ILCs are mainly tissue-resident cells, especially present at the mucosal surface of the intestine and lungs (174). Based on cytokine signature and transcription factors, ILCs can be divided into four groups: ILC1, ILC2, ILC3, and regulatory ILC (ILCreg). ILC1 functions highly overlap with NK cells as both produce IFN-γ, enhancing the ability of macrophages and DCs to remove intracellular pathogens. ILC2s are the innate counterpart of Th2 cells that produce IL-5 and IL-13 and helps in the expulsion of helminths. Notably, type-2 cytokines produced by ILC2s have tissue repair and anti-inflammatory function post-infection (175, 176). ILC3s are the innate counterpart of Th17 cells as they produce IL-17 and IL-22 in response to IL-1β and IL-23. IL-22 can stimulate the secretion of anti-microbial peptides from intestinal cells and provides a barrier in the intestine, whereas IL-17 induces granulopoiesis (177, 178). IL-17 also drives inflammatory response by recruiting cells to the site of inflammation (179).

There are key evidences that suggested the involvement of ILCs in inflammatory bone diseases such as spondylarthritis (SA) (180). It is reported that there is an enrichment in the number of ILC3s in SF of patients with psoriatic arthritis (181). Enrichment of ILC3 is also found in the gut, in the peripheral blood, bone marrow, and SF of patients with ankylosing spondylitis. A recent study has shown that CCR6 positive ILC3s (ILC3^CCR6+^) are enriched in inflamed joints of CIA mice and RA patients and show high IL-17A and IL-22 in arthritic mice (**Figure 4D**) (27). These reports suggested a critical role of ILC3s in the development of all these diseases, probably due to their highly pro-inflammatory nature.

Hirota et al. have reported that GM-CSF-producing ILC2s have a pathogenic role in augmenting arthritis (**Figure 4C**) (41). However, recent studies demonstrated the protective role of ILC2s. They can reduce inflammatory arthritis and prevents bone loss in mice (182). Another study by Omata et al. supported the immune-regulatory role of ILC2s (183). IL-4 and IL-13 secretion from ILC2 trigger STAT6 activation in myeloid cells, resulting in suppression of OC formation, thus preventing OVX-induced bone loss (183). Therefore, ILC2s exert regulatory function on bone homeostasis by impairing osteoclastogenesis. Additionally, ILC2s can have a regulatory effect on bone *via* Treg cells, which are inhibitors of OC formation.

ILC1s are known contributors of IFN-γ and are enriched in many chronic inflammatory diseases. ILC1 is the more predominant ILCs in SF of RA patients (180). ILC1 is the primary subtype of ILCs in gingivitis and periodontitis, and they express RANKL (**Figure 4B**) (42). More descriptive studies on ILC1s expressing RANKL are required to understand their role in bone remodeling.

Recently, a study has recognized a new subset of IL-10 producing ILCs named regulatory ILCs (ILCreg). These are Lin^-^CD45^+^CD127^+^ IL-10^+^ cells and are mostly present in gut tissue (184). In inflammatory conditions, ILCreg can be stimulated in the intestine and acts on other ILCs such as ILC1 and ILC3 to suppress their activation *via* IL-10. Additionally, ILCreg can also produce TGF-β that acts in an autocrine manner for its expansion in inflammatory conditions. Interestingly, IL-10 is a potent anti-inflammatory cytokine that can downregulate the synthesis of pro-inflammatory cytokines such as IL-6, TNF-α, *etc.*, preventing inflammatory-driven osteoclastogenesis and bone resorption (185). Since ILCreg produces IL-10 and suppresses intestinal inflammation, it may also play a role in suppressing inflammatory bone loss. The role of TGF-β in osteoclastogenesis is very complex and controversial, but TGF-β enhances osteoblast proliferation and survival (186). Thus, TGF-β produced by ILCreg may enhance bone formation. Moreover, detailed studies are required to understand the contribution of ILCreg in the suppression of inflammatory disease conditions such as osteoporosis.

## Inflammatory Mediators and Osteoporosis

Some of the key pro-inflammatory mediators secreted by innate immune cells are IL-6, TNF-α, IL-1β, ROS, and IFN-*γ* (1–3, 187, 188).

IL-6 is prominently involved in osteoporosis. An increase in IL-6 in the body induces an increase in osteoclastogenesis *via* the induction of RANKL production from osteoblasts (43). IL-6 upregulates S1PR2 [Sphingosine-1-phosphate (S1P)] receptor on the surface of osteoclast precursor and helps in its transmigration from the bone marrow to the blood and thus play a crucial role in the hallmark systemic bone loss (44). Moreover, two of the inflammatory chemokines CXCL8 and CCL20, enhance osteoblast-induced osteoclastic activity *via* IL-6 production (189).

TNF-α is an important molecule in osteoporotic disorders, especially in post-menopausal osteoporosis (190–193). It acts as pro-osteoporotic either by acting as pro-osteoclastogenic or by impairing osteoblast function. It directly acts on macrophages to increase RANK expression and acts on stromal cells to increase RANKL production (45). TNF-α triggers osteoclastogenesis by synergistically acting with RANKL and M-CSF *via* NF-κB and PI3k/AKT pathway (193). This intensifies the osteoclastic activity by many folds comparing RANKL alone (193). Moreover, another report suggests that TNF-α priming sensitizes M-CSF-induced M2 macrophages to pro-inflammatory M1 macrophage polarization in RelB dependent manner, resulting in expanding osteoclast precursor pool with higher osteoclastic potential (47). TNF-α also induces sclerostin (SOST) expression, which triggers RANKL expression in osteocytes and further enhances osteoclastogenesis (46). Together with IL-6, TNF-α can actively cause osteoclastogenesis independent of RANKL (194).

TNF-α acts as anti-osteogenic by inhibiting differentiation, proliferation, and activities of osteoblast. It upregulates CHIP-ubiquitin ligase protein, which results in the degradation of osterix (pro-osteoblastic transcription factor) (48). TNF-α also inhibits expression of BMP-induced ‘special AT-rich sequence binding protein 2’ (SATB2), which is another pro-osteoblastic transcription factor, by triggering NF-κB binding to SATB2 promoter (49). Further, TNF-α induces upregulation of purinoreceptor P2Y2 through ERK and JNK signaling pathways and hinder the differentiation of MSCs (48, 50). The Canonical WNT/β-catenin pathway is known to regulate bone homeostasis and development. Both IL-6 and TNF-α hamper the pro-osteoblastic WNT/β-catenin pathway by upregulating its antagonists, Dickkopf-related protein 1 (DKK1) and SOST, which prevent osteoblast differentiation (46, 195).

IFN-γ, a type-II interferon, affects later phases of maturation of osteoclasts. An active osteoclast must fuse to form a functional multinucleated osteoclast. This fusion is aided by a transmembrane protein called DC-STAMP, which is expressed by IFN-γ-induced-transcription factors NFATc1 and c-FOS (51). Moreover, IFN-γ-induced upregulation of MHC-II on APCs helps in T-Cell activation. The activated T-Cells produce more TNF-α and RANKL, which further help in the differentiation and maturation of OCs (51).

IL-1β, another highly pro-inflammatory cytokine, promotes RANKL dependent osteoclast differentiation *via* activation of transcription factors NF-κB and AP-1. IL-1β also increases CCR7, which promotes osteoclast migration and activation (52). IL-1β is a prerequisite for the C5a (complement protein)-induced osteoclast activation (53, 54). IL-1β has also been reported to enhance proteolytic enzymes like plasminogen, collagenases, and cathepsin-B, which break down bone matrix proteins resulting in bone loss (55). In addition, it has also been shown to downregulate osteoblastic activities by inhibiting alkaline phosphatase (ALP), which is required for bone mineralization and collagen synthesis activities *via* modulating STATs and SMAD pathways (56).

Reactive oxygen species or ROS, especially hydrogen peroxide and superoxide ions, has been recently shown critical in osteoclast development. ROS has been shown to increase osteoclastic activities and bone loss (196). It has been reported to induce apoptosis in osteoblasts. ROS-activated FOXOs, a subclass of forkhead proteins involved in cell cycle arrest, hinders the WNT/β-catenin pathway in MSCs, thus impairs osteoblastogenesis (197). Moreover, ROS is critical in maintaining body homeostasis, it would be interesting to understand more about the role of ROS in context of osteoporosis.

## Conclusion and Future Perspective

Bone is a complex and dynamic tissue. Bone health depends on multiple factors like diet, age, hormonal, and the inflammatory status of the body. In addition to these factors, osteoporosis is also correlated with age-driven complications in the senile population. A considerable impact of aging has been reported on the immune system and associated pathologies (1, 5). Macrophages, which are the major contributor to initiation and resolution of inflammation, sense the age-related metabolic epigenetic changes and with a constitutive change to M1-type play a major role in ‘Inflammaging’: chronic low-grade inflammation in aged people (10, 83). Thus, our immune system is capable of sensing different stimulus as well as different phases of life and results in pathologies like osteoporosis.

In the past 20 years, the field of osteoimmunology allowed us to appreciate the underlying mechanisms of different bone pathologies by integrating the knowledge from the immune system and bone biology. Such studies have provided new insights into how both the system functions in a concerted manner to carry out a complex process of bone modeling and remodeling. The advent of the new field of “immunoporosis” emphasizes the role of immune system players in the pathophysiology of osteoporosis. As discussed in the review, several innate immune cells have emerged as key regulators of immunoporosis. These innate immune cells modulate osteoporosis by producing several pro-inflammatory mediators and *via* modulation of cells important for causing osteoporosis largely by affecting the RANK/RANKL/OPG axis. Net bone destructive activity of osteoclasts seemingly is the decisive factor manifesting in bone status. Moreover, the fact that OCs and some major innate immune cells share a common origin as well as developmental niche, allow them to carry various overlapping features such as expression of common array of PRRs, production of various pro-inflammatory cytokines and their receptors, creating an efficient nexus of information between skeletal and immune system. That is how immune system senses the physiological status of the body and controls the skeletal system. Therefore, research towards this can allow us to find more therapeutic molecular targets to tackle osteoporosis. In addition to the innate immune cells discussed above, inflammation-mediated by intestinal epithelial cells, B1 cells, *γ*δ T cells could play an important role in osteoporosis, and further study on these cells could be intriguing.

Additionally, in the past few years, there has been increasing evidence linking gut microbiota with bone health. It is now known that the gut controls several inflammatory diseases by the cross-talk between the innate immune cells and gut-microbiota (198, 199). Further, studying the cross talk between gut microbiota and ILCs and intestinal cells could be important in immunoporosis and of great clinical value.

## Author Contributions

YS and SR wrote the manuscript, and prepared the figures and table. AM conceived the idea, organized the overall design of the manuscript, and wrote the manuscript. All authors contributed to the article and approved the submitted version.

## Funding

Funding from Indian Institute of Science Education and Research Mohali.

## Conflict of Interest

The authors declare that the research was conducted in the absence of any commercial or financial relationships that could be construed as a potential conflict of interest.

## Publisher’s Note

All claims expressed in this article are solely those of the authors and do not necessarily represent those of their affiliated organizations, or those of the publisher, the editors and the reviewers. Any product that may be evaluated in this article, or claim that may be made by its manufacturer, is not guaranteed or endorsed by the publisher.

## Glossary

**Table:**

ADCC	Antibody-mediated cell cytotoxicity
APCs	Antigen-presenting cells
BMD	Bone mineral density
BMDMs	Bone marrow-derived macrophages
BMMs	Bone marrow macrophages
BMP	Morphogenetic protein
BRC	Bone Remodelling Compartment
CCR2	C-C chemokine receptor 2
CIA	Collagen-induced arthritis
cMoP	Committed monocyte progenitor
COPD	Chronic obstructive pulmonary disease
CSF1	Colony stimulating factor 1
CX3CR1	CXC3 chemokine receptor 1
DAXX	Death-associated protein 6
DC	Dendritic cells
DNAM-1	DNAX accessory molecule-1
EoE	Eosinophilic esophagitis
GCR	Glucocorticoid receptor
HDC	Histidine decarboxylase
HSCs	Hematopoietic stem cells
HSP27	Heat-shock protein 27
IFN	Interferon
IkBα	Inhibitor of NF-kB
ILCs	Innate lymphoid cells
LAD-I	Leukocyte adhesion deficiency type-I
LFA-1	Leucocyte function-associated antigen-1
MAPKs	Mitogen-activated protein kinase
M-CSF	Macrophage colony stimulating factor
MHC II	Major histocompatibility factor II
MSCs	Mesenchymal stem cells
NET	Neutrophils extracellular traps
NFATc	Nuclear factor of activated T-Cells
NLRs	Nod-like receptors
OA	Osteoarthritis
OBs	Osteoblasts
OCs	Osteoclasts
OPG	Osteoprotegerin
OVX	Ovariectomized
OYs	Osteocytes
PBM	Peripheral blood monocytes
PBMCs	Peripheral blood mononuclear cells
pDCs	Plasmacytoid DCs
PI3K	Phosphoinositide 3-kinase
PLK3	Polo-like kinase 3
PMN	Polymorphonuclear
PRRs	Pattern recognition receptors
RA	Rheumatoid arthritis
RANK	Receptor activator of nuclear factor-κB
RANKL	Receptor activator of nuclear factor-κB ligand
ROS	Reactive oxygen species
SDS	Shwachman-Diamond syndrome showing skeletal defects
SF	Synovial fluid
SRs	Scavenger receptors
STAT1	Signal transducer and activator of transcription
TLRs	Toll-like receptors
TNF	Tumor necrosis factor
RAF	TNF receptor-associated factor
TRAP	Tartrate-resistant acid phosphatase

## References

[1] Clowes JA Riggs BL Khosla S. The Role of the Immune System in the Pathophysiology of Osteoporosis. Immunol Rev (2005) 208:207–27. 10.1111/j.0105-2896.2005.00334.x 16313351

[2] Okman-Kilic T. Estrogen Deficiency and Osteoporosis. Advances in Osteoporosis. Yannis Dionyssiotis. IntechOpen (2015). 10.5772/59407

[3] Walsh MC Takegahara N Kim H Choi Y. Updating Osteoimmunology: Regulation of Bone Cells by Innate and Adaptive Immunity. Nat Rev Rheumatol (2018) 14(3):146–56. 10.1038/nrrheum.2017.213 PMC5821527 29323344

[4] Tsukasaki M Takayanagi H. Osteoimmunology: Evolving Concepts in Bone-Immune Interactions in Health and Disease. Nat Rev Immunol (2019) 19(10):626–42. 10.1038/s41577-019-0178-8 31186549

[5] Ferrucci L Fabbri E. Inflammageing: Chronic Inflammation in Ageing, Cardiovascular Disease, and Frailty. Nat Rev Cardiol (2018) 15(9):505–22. 10.1038/s41569-018-0064-2 PMC6146930 30065258

[6] Horton JE Raisz LG Simmons HA Oppenheim JJ Mergenhagen SE. Bone Resorbing Activity in Supernatant Fluid From Cultured Human Peripheral Blood Leukocytes. Science (1972) 177(4051):793–5. 10.1126/science.177.4051.793 5052733

[7] Mundy GR Luben RA Raisz LG Oppenheim JJ Buell DN. Bone-Resorbing Activity in Supernatants From Lymphoid Cell Lines. New Engl J Med (1974) 290(16):867–71. 10.1056/NEJM197404182901601 4816960

[8] Takayanagi H Ogasawara K Hida S Chiba T Murata S Sato K. T-Cell-Mediated Regulation of Osteoclastogenesis by Signalling Cross-Talk Between RANKL and IFN-Gamma. Nature (2000) 408(6812):600–5. 10.1038/35046102 11117749

[9] Wong BR Josien R Choi Y. TRANCE is a TNF Family Member That Regulates Dendritic Cell and Osteoclast Function. J Leukocyte Biol (1999) 65(6):715–24. 10.1002/jlb.65.6.715 10380891

[10] Yarbro JR Emmons RS Pence BD. Macrophage Immunometabolism and Inflammaging: Roles of Mitochondrial Dysfunction, Cellular Senescence, CD38, and NAD. Immunometabolism (2020) 2(3):e200026. 10.20900/immunometab20200026 32774895 PMC7409778

[11] Arron JR Choi Y. Bone *Versus* Immune System. Nature (2000) 408(6812):535–6. 10.1038/35046196 11117729

[12] Srivastava RK Dar HY Mishra PK. Immunoporosis: Immunology of Osteoporosis-Role of T Cells. Front Immunol (2018) 9:657. 10.3389/fimmu.2018.00657 29675022 PMC5895643

[13] Sapra L Azam Z Rani L Saini C Bhardwaj A Shokeen N. “Immunoporosis”: Immunology of Osteoporosis. Proc Natl Acad Sci India Section B: Biol Sci (2021) 1–9. 10.1007/s40011-021-01238-x

[14] Hardy R Cooper MS. Bone Loss in Inflammatory Disorders. J Endocrinol (2009) 201(3):309–20. 10.1677/JOE-08-0568 19443863

[15] Hato T Dagher PC. How the Innate Immune System Senses Trouble and Causes Trouble. Clin J Am Soc Nephrology: CJASN (2015) 10(8):1459–69. 10.2215/CJN.04680514 PMC4527020 25414319

[16] Tao Z Wang J Wen K Yao R Da W Zhou S. Pyroptosis in Osteoblasts: A Novel Hypothesis Underlying the Pathogenesis of Osteoporosis. Front Endocrinol (2020) 11:548812. 10.3389/fendo.2020.548812 PMC7821870 33488513

[17] Adami G Saag KG. Osteoporosis Pathophysiology, Epidemiology, and Screening in Rheumatoid Arthritis. Curr Rheumatol Rep (2019) 21(7):34. 10.1007/s11926-019-0836-7 31123839

[18] Cobo G Lindholm B Stenvinkel P. Chronic Inflammation in End-Stage Renal Disease and Dialysis Vol. 33. Nephrology, dialysis, transplantation: official publication of the European Dialysis and Transplant Association - European Renal Association (2018) p. iii35–40. 10.1093/ndt/gfy175 PMC6168801 30281126

[19] Munoz-Torres M Aguado P Dauden E Carrascosa JM Rivera R. Osteoporosis and Psoriasis. Actas dermo-sifiliograficas (2019) 110(8):642–52. 10.1016/j.ad.2019.02.005 31151668

[20] Wang CJ McCauley LK. Osteoporosis and Periodontitis. Curr Osteoporosis Rep (2016) 14(6):284–91. 10.1007/s11914-016-0330-3 PMC5654540 27696284

[21] Ponzetti M Rucci N. Updates on Osteoimmunology: What’s New on the Cross-Talk Between Bone and Immune System. Front Endocrinol (2019) 10:236. 10.3389/fendo.2019.00236 PMC6482259 31057482

[22] Quinn JM Neale S Fujikawa Y McGee JO Athanasou NA. Human Osteoclast Formation From Blood Monocytes, Peritoneal Macrophages, and Bone Marrow Cells. Calcified Tissue Int (1998) 62(6):527–31. 10.1007/s002239900473 9576981

[23] Yu XY Li XS Li Y Liu T Wang RT. Neutrophil-Lymphocyte Ratio Is Associated With Arterial Stiffness in Postmenopausal Women With Osteoporosis. Arch GerontoL Geriatrics (2015) 61(1):76–80. 10.1016/j.archger.2015.03.011 25882272

[24] Hu X Sun Y Xu W Lin T Zeng H. Expression of RANKL by Peripheral Neutrophils and its Association With Bone Mineral Density in COPD. Respirology (2017) 22(1):126–32. 10.1111/resp.12878 27552066

[25] Ragipoglu D Dudeck A Haffner-Luntzer M Voss M Kroner J Ignatius A. The Role of Mast Cells in Bone Metabolism and Bone Disorders. Front Immunol (2020) 11:163. 10.3389/fimmu.2020.00163 32117297 PMC7025484

[26] Soderstrom K Stein E Colmenero P Purath U Muller-Ladner U de Matos CT. Natural Killer Cells Trigger Osteoclastogenesis and Bone Destruction in Arthritis. Proc Natl Acad Sci USA (2010) 107(29):13028–33. 10.1073/pnas.1000546107 PMC2919936 20615964

[27] Takaki-Kuwahara A Arinobu Y Miyawaki K Yamada H Tsuzuki H Irino K. CCR6+ Group 3 Innate Lymphoid Cells Accumulate in Inflamed Joints in Rheumatoid Arthritis and Produce Th17 Cytokines. Arthritis Res Ther (2019) 21(1):198. 10.1186/s13075-019-1984-x 31470891 PMC6716915

[28] Lassus J Salo J Jiranek WA Santavirta S Nevalainen J Matucci-Cerinic M. Macrophage Activation Results in Bone Resorption. Clin Orthopaedics Related Res (1998) 352):7–15. 10.1097/00003086-199807000-00003 9678028

[29] Gong L Zhao Y Zhang Y Ruan Z. The Macrophage Polarization Regulates MSC Osteoblast Differentiation *In Vitro* . Ann Clin Lab Sci (2016) 46(1):65–71. 26927345

[30] Zhang Y Bose T Unger RE Jansen JA Kirkpatrick CJ van den Beucken J. Macrophage Type Modulates Osteogenic Differentiation of Adipose Tissue MSCs. Cell Tissue Res (2017) 369(2):273–86. 10.1007/s00441-017-2598-8 PMC5552848 28361303

[31] Dou C Ding N Zhao C Hou T Kang F Cao Z. Estrogen Deficiency-Mediated M2 Macrophage Osteoclastogenesis Contributes to M1/M2 Ratio Alteration in Ovariectomized Osteoporotic Mice. J Bone Mineral Research: Off J Am Soc Bone Mineral Res (2018) 33(5):899–908. 10.1002/jbmr.3364 29281118

[32] Chang MK Raggatt LJ Alexander KA Kuliwaba JS Fazzalari NL Schroder K. Osteal Tissue Macrophages are Intercalated Throughout Human and Mouse Bone Lining Tissues and Regulate Osteoblast Function *In Vitro* and *In Vivo* . J Immunol (2008) 181(2):1232–44. 10.4049/jimmunol.181.2.1232 18606677

[33] Sprangers S de Vries TJ Everts V. Monocyte Heterogeneity: Consequences for Monocyte-Derived Immune Cells. J Immunol Res (2016) 2016:1475435. 10.1155/2016/1475435 27478854 PMC4958468

[34] Gebraad A Kornilov R Kaur S Miettinen S Haimi S Peltoniemi H. Monocyte-Derived Extracellular Vesicles Stimulate Cytokine Secretion and Gene Expression of Matrix Metalloproteinases by Mesenchymal Stem/Stromal Cells. FEBS J (2018) 285(12):2337–59. 10.1111/febs.14485 29732732

[35] Rivollier A Mazzorana M Tebib J Piperno M Aitsiselmi T Rabourdin-Combe C. Immature Dendritic Cell Transdifferentiation Into Osteoclasts: A Novel Pathway Sustained by the Rheumatoid Arthritis Microenvironment. Blood (2004) 104(13):4029–37. 10.1182/blood-2004-01-0041 15308576

[36] Chakravarti A Raquil MA Tessier P Poubelle PE. Surface RANKL of Toll-Like Receptor 4-Stimulated Human Neutrophils Activates Osteoclastic Bone Resorption. Blood (2009) 114(8):1633–44. 10.1182/blood-2008-09-178301 19546479

[37] Sirufo MM Suppa M Ginaldi L De Martinis M. Does Allergy Break Bones? Osteoporosis and Its Connection to Allergy. Int J Mol Sci (2020) 21(3):712. 10.3390/ijms21030712 PMC7037724 31973226

[38] Ginaldi L De Martinis M Ciccarelli F Saitta S Imbesi S Mannucci C. Increased Levels of Interleukin 31 (IL-31) in Osteoporosis. BMC Immunol (2015) 16:60. 10.1186/s12865-015-0125-9 26449657 PMC4599585

[39] Buckley MG Walters C Wong WM Cawley MI Ren S Schwartz LB. Mast Cell Activation in Arthritis: Detection of Alpha- and Beta-Tryptase, Histamine and Eosinophil Cationic Protein in Synovial Fluid. Clin Sci (1997) 93(4):363–70. 10.1042/cs0930363 9404229

[40] Malone DG Irani AM Schwartz LB Barrett KE Metcalfe DD. Mast Cell Numbers and Histamine Levels in Synovial Fluids From Patients With Diverse Arthritides. Arthritis Rheum (1986) 29(8):956–63. 10.1002/art.1780290803 2427093

[41] Hirota K Hashimoto M Ito Y Matsuura M Ito H Tanaka M. Autoimmune Th17 Cells Induced Synovial Stromal and Innate Lymphoid Cell Secretion of the Cytokine GM-CSF to Initiate and Augment Autoimmune Arthritis. Immunity (2018) 48(6):1220–32.e5. 10.1016/j.immuni.2018.04.009 29802020 PMC6024031

[42] Kindstedt E Koskinen Holm C Palmqvist P Sjostrom M Lejon K Lundberg P. Innate Lymphoid Cells are Present in Gingivitis and Periodontitis. J Periodontol (2019) 90(2):200–7. 10.1002/JPER.17-0750 30070705

[43] Wang T He C. TNF-Alpha and IL-6: The Link Between Immune and Bone System. Curr Drug Targets (2020) 21(3):213–27. 10.2174/1389450120666190821161259 31433756

[44] Tanaka K Hashizume M Mihara M Yoshida H Suzuki M Matsumoto Y. Anti-Interleukin-6 Receptor Antibody Prevents Systemic Bone Mass Loss *via* Reducing the Number of Osteoclast Precursors in Bone Marrow in a Collagen-Induced Arthritis Model. Clin Exp Immunol (2014) 175(2):172–80. 10.1111/cei.12201 PMC3892408 24028747

[45] Luo G Li F Li X Wang ZG Zhang B. TNFalpha and RANKL Promote Osteoclastogenesis by Upregulating RANK *via* the NFkappaB Pathway. Mol Med Rep (2018) 17(5):6605–11. 10.3892/mmr.2018.8698 PMC5928634 29512766

[46] Ohori F Kitaura H Marahleh A Kishikawa A Ogawa S Qi J. Effect of TNF-Alpha-Induced Sclerostin on Osteocytes During Orthodontic Tooth Movement. J Immunol Res (2019) 2019:9716758. 10.1155/2019/9716758 31341915 PMC6612957

[47] Zhao Z Hou X Yin X Li Y Duan R Boyce BF. TNF Induction of NF-kappaB RelB Enhances RANKL-Induced Osteoclastogenesis by Promoting Inflammatory Macrophage Differentiation But Also Limits It Through Suppression of NFATc1 Expression. PloS One (2015) 10(8):e0135728. 10.1371/journal.pone.0135728 26287732 PMC4545392

[48] Xie J Gu J. Identification of C-Terminal Hsp70-Interacting Protein as a Mediator of Tumour Necrosis Factor Action in Osteoblast Differentiation by Targeting Osterix for Degradation. J Cell Mol Med (2015) 19(8):1814–24. 10.1111/jcmm.12553 PMC4549032 25818514

[49] Zuo C Zhao X Shi Y Wu W Zhang N Xu J. TNF-Alpha Inhibits SATB2 Expression and Osteoblast Differentiation Through NF-kappaB and MAPK Pathways. Oncotarget (2018) 9(4):4833–50. 10.18632/oncotarget.23373 PMC5797016 29435145

[50] Du D Zhou Z Zhu L Hu X Lu J Shi C. TNF-Alpha Suppresses Osteogenic Differentiation of MSCs by Accelerating P2Y2 Receptor in Estrogen-Deficiency Induced Osteoporosis. Bone (2018) 117:161–70. 10.1016/j.bone.2018.09.012 30236554

[51] Tang M Tian L Luo G Yu X. Interferon-Gamma-Mediated Osteoimmunology. Front Immunol (2018) 9:1508. 10.3389/fimmu.2018.01508 30008722 PMC6033972

[52] Lee J Park C Kim HJ Lee YD Lee ZH Song YW. Stimulation of Osteoclast Migration and Bone Resorption by C-C Chemokine Ligands 19 and 21. Exp Mol Med (2017) 49(7):e358. 10.1038/emm.2017.100 28729639 PMC5565950

[53] Ignatius A Schoengraf P Kreja L Liedert A Recknagel S Kandert S. Complement C3a and C5a Modulate Osteoclast Formation and Inflammatory Response of Osteoblasts in Synergism With IL-1beta. J Cell Biochem (2011) 112(9):2594–605. 10.1002/jcb.23186 PMC3158833 21598302

[54] Pobanz JM Reinhardt RA Koka S Sanderson SD. C5a Modulation of Interleukin-1 Beta-Induced Interleukin-6 Production by Human Osteoblast-Like Cells. J Periodontal Res (2000) 35(3):137–45. 10.1034/j.1600-0765.2000.035003137.x 10929868

[55] Panagakos FS Jandinski JJ Feder L Kumar S. Effects of Plasminogen and Interleukin-1 Beta on Bone Resorption *In Vitro* . Biochimie (1994) 76(5):394–7. 10.1016/0300-9084(94)90114-7 7849104

[56] Ruscitti P Cipriani P Carubbi F Liakouli V Zazzeroni F Di Benedetto P. The Role of IL-1beta in the Bone Loss During Rheumatic Diseases. Mediators Inflamm (2015) 2015:782382. 10.1155/2015/782382 25954061 PMC4410538

[57] Mukhopadhyay S Pluddemann A Gordon S. Macrophage Pattern Recognition Receptors in Immunity, Homeostasis and Self Tolerance. Adv Exp Med Biol (2009) 653:1–14. 10.1007/978-1-4419-0901-5_1 19799108 PMC7123833

[58] Alonso-Perez A Franco-Trepat E Guillan-Fresco M Jorge-Mora A Lopez V Pino J. Role of Toll-Like Receptor 4 on Osteoblast Metabolism and Function. Front Physiol (2018) 9:504. 10.3389/fphys.2018.00504 29867550 PMC5952219

[59] Kassem A Lindholm C Lerner UH. Toll-Like Receptor 2 Stimulation of Osteoblasts Mediates Staphylococcus Aureus Induced Bone Resorption and Osteoclastogenesis Through Enhanced RANKL. PloS One (2016) 11(6):e0156708. 10.1371/journal.pone.0156708 27311019 PMC4911171

[60] Koduru SV Sun BH Walker JM Zhu M Simpson C Dhodapkar M. The Contribution of Cross-Talk Between the Cell-Surface Proteins CD36 and CD47-TSP-1 in Osteoclast Formation and Function. J Biol Chem (2018) 293(39):15055–69. 10.1074/jbc.RA117.000633 PMC6166722 30082316

[61] Li X Wang X Hu Z Chen Z Li H Liu X. Possible Involvement of the oxLDL/LOX-1 System in the Pathogenesis and Progression of Human Intervertebral Disc Degeneration or Herniation. Sci Rep (2017) 7(1):7403. 10.1038/s41598-017-07780-x 28785062 PMC5547039

[62] Michalski MN McCauley LK. Macrophages and Skeletal Health. Pharmacol Ther (2017) 174:43–54. 10.1016/j.pharmthera.2017.02.017 28185913 PMC5429177

[63] Sica A Mantovani A. Macrophage Plasticity and Polarization: *In Vivo* Veritas. J Clin Invest (2012) 122(3):787–95. 10.1172/JCI59643 PMC3287223 22378047

[64] Liu H Wu X Gang N Wang S Deng W Zan L. Macrophage Functional Phenotype can be Consecutively and Reversibly Shifted to Adapt to Microenvironmental Changes. Int J Clin Exp Med (2015) 8(2):3044–53. PMC4402928 25932281

[65] Murray PJ. Macrophage Polarization. Annu Rev Physiol (2017) 79:541–66. 10.1146/annurev-physiol-022516-034339 27813830

[66] Huang R Wang X Zhou Y Xiao Y. RANKL-Induced M1 Macrophages are Involved in Bone Formation. Bone Res (2017) 5:17019. 10.1038/boneres.2017.19 29263936 PMC5645773

[67] Loi F Cordova LA Zhang R Pajarinen J Lin TH Goodman SB. The Effects of Immunomodulation by Macrophage Subsets on Osteogenesis *In Vitro* . Stem Cell Res Ther (2016) 7:15. 10.1186/s13287-016-0276-5 26801095 PMC4724110

[68] Champagne CM Takebe J Offenbacher S Cooper LF. Macrophage Cell Lines Produce Osteoinductive Signals That Include Bone Morphogenetic Protein-2. Bone (2002) 30(1):26–31. 10.1016/S8756-3282(01)00638-X 11792561

[69] Takito J Nakamura M. Heterogeneity and Actin Cytoskeleton in Osteoclast and Macrophage Multinucleation. Int J Mol Sci (2020) 21(18):6629. 10.3390/ijms21186629 PMC7554939 32927783

[70] Feng W Guo J Li M. RANKL-Independent Modulation of Osteoclastogenesis. J Oral Biosci (2019) 61(1):16–21. 10.1016/j.job.2019.01.001 30929797

[71] Bordbar A Mo ML Nakayasu ES Schrimpe-Rutledge AC Kim YM Metz TO. Model-Driven Multi-Omic Data Analysis Elucidates Metabolic Immunomodulators of Macrophage Activation. Mol Syst Biol (2012) 8:558. 10.1038/msb.2012.21 22735334 PMC3397418

[72] An E Narayanan M Manes NP Nita-Lazar A. Characterization of Functional Reprogramming During Osteoclast Development Using Quantitative Proteomics and mRNA Profiling. Mol Cell Proteomics: MCP (2014) 13(10):2687–704. 10.1074/mcp.M113.034371 PMC4188996 25044017

[73] Park-Min KH. Metabolic Reprogramming in Osteoclasts. Semin Immunopathol (2019) 41(5):565–72. 10.1007/s00281-019-00757-0 PMC7671717 31552471

[74] Kim JM Jeong D Kang HK Jung SY Kang SS Min BM. Osteoclast Precursors Display Dynamic Metabolic Shifts Toward Accelerated Glucose Metabolism at an Early Stage of RANKL-Stimulated Osteoclast Differentiation. Cell Physiol Biochem: Int J Exp Cell Physiol Biochem Pharmacol (2007) 20(6):935–46. 10.1159/000110454 17982276

[75] Yao Y Cai X Ren F Ye Y Wang F Zheng C. The Macrophage-Osteoclast Axis in Osteoimmunity and Osteo-Related Diseases. Front Immunol (2021) 12:664871. 10.3389/fimmu.2021.664871 33868316 PMC8044404

[76] Gu Q Yang H Shi Q. Macrophages and Bone Inflammation. J Orthopaedic Transl (2017) 10:86–93. 10.1016/j.jot.2017.05.002 PMC5822954 29662760

[77] Yang X Chang Y Wei W. Emerging Role of Targeting Macrophages in Rheumatoid Arthritis: Focus on Polarization, Metabolism and Apoptosis. Cell Prolif (2020) 53(7):e12854. 10.1111/cpr.12854 32530555 PMC7377929

[78] Wu CL Harasymowicz NS Klimak MA Collins KH Guilak F. The Role of Macrophages in Osteoarthritis and Cartilage Repair. Osteoarthritis Cartilage (2020) 28(5):544–54. 10.1016/j.joca.2019.12.007 PMC7214213 31926267

[79] Chen G Xu Q Dai M Liu X. Bergapten Suppresses RANKL-Induced Osteoclastogenesis and Ovariectomy-Induced Osteoporosis *via* Suppression of NF-kappaB and JNK Signaling Pathways. Biochem Biophys Res Commun (2019) 509(2):329–34. 10.1016/j.bbrc.2018.12.112 30579598

[80] Kim B Lee KY Park B. Icariin Abrogates Osteoclast Formation Through the Regulation of the RANKL-Mediated TRAF6/NF-Kappab/ERK Signaling Pathway in Raw264.7 Cells. Phytomedicine: Int J Phytother Phytopharmacol (2018) 51:181–90. 10.1016/j.phymed.2018.06.020 30466615

[81] Choo YY Tran PT Min BS Kim O Nguyen HD Kwon SH. Sappanone A Inhibits RANKL-Induced Osteoclastogenesis in BMMs and Prevents Inflammation-Mediated Bone Loss. Int Immunopharmacol (2017) 52:230–7. 10.1016/j.intimp.2017.09.018 28946117

[82] Matsumoto M Sudo T Saito T Osada H Tsujimoto M. Involvement of P38 Mitogen-Activated Protein Kinase Signaling Pathway in Osteoclastogenesis Mediated by Receptor Activator of NF-kappa B Ligand (RANKL). J Biol Chem (2000) 275(40):31155–61. 10.1074/jbc.M001229200 10859303

[83] Stahl EC Haschak MJ Popovic B Brown BN. Macrophages in the Aging Liver and Age-Related Liver Disease. Front Immunol (2018) 9:2795. 10.3389/fimmu.2018.02795 30555477 PMC6284020

[84] Kim OH Kim H Kang J Yang D Kang YH Lee DH. Impaired Phagocytosis of Apoptotic Cells Causes Accumulation of Bone Marrow-Derived Macrophages in Aged Mice. BMB Rep (2017) 50(1):43–8. 10.5483/BMBRep.2017.50.1.167 PMC5319664 27866511

[85] Barrett JP Costello DA O’Sullivan J Cowley TR Lynch MA. Bone Marrow-Derived Macrophages From Aged Rats are More Responsive to Inflammatory Stimuli. J Neuroinflamm (2015) 12:67. 10.1186/s12974-015-0287-7 PMC4397943 25890218

[86] Smallwood HS Lopez-Ferrer D Squier TC. Aging Enhances the Production of Reactive Oxygen Species and Bactericidal Activity in Peritoneal Macrophages by Upregulating Classical Activation Pathways. Biochemistry (2011) 50(45):9911–22. 10.1021/bi2011866 PMC3241613 21981794

[87] Clark D Brazina S Yang F Hu D Hsieh CL Niemi EC. Age-Related Changes to Macrophages Are Detrimental to Fracture Healing in Mice. Aging Cell (2020) 19(3):e13112. 10.1111/acel.13112 32096907 PMC7059136

[88] Gibon E Loi F Cordova LA Pajarinen J Lin T Lu L. Aging Affects Bone Marrow Macrophage Polarization: Relevance to Bone Healing. Regenerative Eng Trans Med (2016) 2(2):98–104. 10.1007/s40883-016-0016-5 PMC5270653 28138512

[89] Chen X Ouyang Z Shen Y Liu B Zhang Q Wan L. CircRNA_28313/miR-195a/CSF1 Axis Modulates Osteoclast Differentiation to Affect OVX-Induced Bone Absorption in Mice. RNA Biol (2019) 16(9):1249–62. 10.1080/15476286.2019.1624470 PMC6693548 31204558

[90] Shen G Ren H Shang Q Zhang Z Zhao W Yu X. miR-128 Plays a Critical Role in Murine Osteoclastogenesis and Estrogen Deficiency-Induced Bone Loss. Theranostics (2020) 10(10):4334–48. 10.7150/thno.42982 PMC7150474 32292498

[91] Dinesh P Kalaiselvan S Sujitha S Rasool M. miR-506-3p Alleviates Uncontrolled Osteoclastogenesis *via* Repression of RANKL/NFATc1 Signaling Pathway. J Cell Physiol (2020) 235(12):9497–509. 10.1002/jcp.29757 32372426

[92] Italiani P Boraschi D. From Monocytes to M1/M2 Macrophages: Phenotypical vs. Functional Differentiation. Front Immunol (2014) 5:514. 10.3389/fimmu.2014.00514 25368618 PMC4201108

[93] Auffray C Fogg D Garfa M Elain G Join-Lambert O Kayal S. Monitoring of Blood Vessels and Tissues by a Population of Monocytes With Patrolling Behavior. Science (2007) 317(5838):666–70. 10.1126/science.1142883 17673663

[94] Tsou CL Peters W Si Y Slaymaker S Aslanian AM Weisberg SP. Critical Roles for CCR2 and MCP-3 in Monocyte Mobilization From Bone Marrow and Recruitment to Inflammatory Sites. J Clin Invest (2007) 117(4):902–9. 10.1172/JCI29919 PMC1810572 17364026

[95] Auffray C Sieweke MH Geissmann F. Blood Monocytes: Development, Heterogeneity, and Relationship With Dendritic Cells. Annu Rev Immunol (2009) 27:669–92. 10.1146/annurev.immunol.021908.132557 19132917

[96] Kratofil RM Kubes P Deniset JF. Monocyte Conversion During Inflammation and Injury. Arteriosc Thromb Vasc Biol (2017) 37(1):35–42. 10.1161/ATVBAHA.116.308198 27765768

[97] Jakubzick C Gautier EL Gibbings SL Sojka DK Schlitzer A Johnson TE. Minimal Differentiation of Classical Monocytes as They Survey Steady-State Tissues and Transport Antigen to Lymph Nodes. Immunity (2013) 39(3):599–610. 10.1016/j.immuni.2013.08.007 24012416 PMC3820017

[98] Yahara Y Barrientos T Tang YJ Puviindran V Nadesan P Zhang H. Erythromyeloid Progenitors Give Rise to a Population of Osteoclasts That Contribute to Bone Homeostasis and Repair. Nat Cell Biol (2020) 22(1):49–59. 10.1038/s41556-019-0437-8 31907410 PMC6953622

[99] Yahara Y Ma X Gracia L Alman BA. Monocyte/Macrophage Lineage Cells From Fetal Erythromyeloid Progenitors Orchestrate Bone Remodeling and Repair. Front Cell Dev Biol (2021) 9(123):1–16. 10.3389/fcell.2021.622035 PMC7889961 33614650

[100] Jacome-Galarza CE Percin GI Muller JT Mass E Lazarov T Eitler J. Developmental Origin, Functional Maintenance and Genetic Rescue of Osteoclasts. Nature (2019) 568(7753):541–5. 10.1038/s41586-019-1105-7 PMC6910203 30971820

[101] Da W Tao L Zhu Y. The Role of Osteoclast Energy Metabolism in the Occurrence and Development of Osteoporosis. Front Endocrinol (2021) 12(556):1–18. 10.3389/fendo.2021.675385 PMC8150001 34054735

[102] Schmidl C Renner K Peter K Eder R Lassmann T Balwierz PJ. Transcription and Enhancer Profiling in Human Monocyte Subsets. Blood (2014) 123(17):e90–9. 10.1182/blood-2013-02-484188 24671955

[103] Zhang L Liu YZ Zeng Y Zhu W Zhao YC Zhang JG. Network-Based Proteomic Analysis for Postmenopausal Osteoporosis in Caucasian Females. Proteomics (2016) 16(1):12–28. 10.1002/pmic.201500005 26435169

[104] Deng FY Lei SF Zhang Y Zhang YL Zheng YP Zhang LS. Peripheral Blood Monocyte-Expressed ANXA2 Gene is Involved in Pathogenesis of Osteoporosis in Humans. Mol Cell Proteomics: MCP (2011) 10(11):M111 011700. 10.1074/mcp.M111.011700 PMC3226411 21817168

[105] Liu YZ Dvornyk V Lu Y Shen H Lappe JM Recker RR. A Novel Pathophysiological Mechanism for Osteoporosis Suggested by an *In Vivo* Gene Expression Study of Circulating Monocytes. J Biol Chem (2005) 280(32):29011–6. 10.1074/jbc.M501164200 15965235

[106] Chen XD Xiao P Lei SF Liu YZ Guo YF Deng FY. Gene Expression Profiling in Monocytes and SNP Association Suggest the Importance of the STAT1 Gene for Osteoporosis in Both Chinese and Caucasians. J Bone Mineral Research: Off J Am Soc Bone Mineral Res (2010) 25(2):339–55. 10.1359/jbmr.090724 PMC3153389 19594299

[107] Daswani B Gupta MK Gavali S Desai M Sathe GJ Patil A. Monocyte Proteomics Reveals Involvement of Phosphorylated HSP27 in the Pathogenesis of Osteoporosis. Dis Markers (2015) 2015:196589. 10.1155/2015/196589 26063949 PMC4439496

[108] Liu YZ Zhou Y Zhang L Li J Tian Q Zhang JG. Attenuated Monocyte Apoptosis, a New Mechanism for Osteoporosis Suggested by a Transcriptome-Wide Expression Study of Monocytes. PloS One (2015) 10(2):e0116792. 10.1371/journal.pone.0116792 25659073 PMC4319757

[109] Leung R Cuddy K Wang Y Rommens J Glogauer M. Sbds is Required for Rac2-Mediated Monocyte Migration and Signaling Downstream of RANK During Osteoclastogenesis. Blood (2011) 117(6):2044–53. 10.1182/blood-2010-05-282574 21084708

[110] Leung R Wang Y Cuddy K Sun C Magalhaes J Grynpas M. Filamin A Regulates Monocyte Migration Through Rho Small GTPases During Osteoclastogenesis. J Bone Mineral Res (2010) 25(5):1077–91. 10.1359/jbmr.091114 19929439

[111] Swiecki M Colonna M. The Multifaceted Biology of Plasmacytoid Dendritic Cells. Nat Rev Immunol (2015) 15(8):471–85. 10.1038/nri3865 PMC4808588 26160613

[112] Maitra R Follenzi A Yaghoobian A Montagna C Merlin S Cannizzo ES. Dendritic Cell-Mediated *In Vivo* Bone Resorption. J Immunol (2010) 185(3):1485–91. 10.4049/jimmunol.0903560 PMC3652267 20581147

[113] Gillespie MT. Impact of Cytokines and T Lymphocytes Upon Osteoclast Differentiation and Function. Arthritis Res Ther (2007) 9(2):103. 10.1186/ar2141 17381830 PMC1906805

[114] Sarkar S Fox DA. Dendritic Cells in Rheumatoid Arthritis. Front Biosci: J Virtual Library (2005) 10:656–65. 10.2741/1560 15569606

[115] Teng YT. Protective and Destructive Immunity in the Periodontium: Part 2–T-Cell-Mediated Immunity in the Periodontium. J Dental Res (2006) 85(3):209–19. 10.1177/154405910608500302 16498066

[116] Cline-Smith A Axelbaum A Shashkova E Chakraborty M Sanford J Panesar P. Ovariectomy Activates Chronic Low-Grade Inflammation Mediated by Memory T Cells, Which Promotes Osteoporosis in Mice. J Bone Mineral Research: Off J Am Soc Bone Mineral Res (2020) 35(6):1174–87. 10.1002/jbmr.3966 PMC8061311 31995253

[117] Wang B Dong Y Tian Z Chen Y Dong S. The Role of Dendritic Cells Derived Osteoclasts in Bone Destruction Diseases. Genes Dis (2020) 8(4):401–11. 10.1016/j.gendis.2020.03.009 PMC8209356 34179305

[118] Narisawa M Kubo S Okada Y Yamagata K Nakayamada S Sakata K. Human Dendritic Cell-Derived Osteoclasts With High Bone Resorption Capacity and T Cell Stimulation Ability. Bone (2021) 142:115616. 10.1016/j.bone.2020.115616 32866681

[119] Alnaeeli M Penninger JM Teng YT. Immune Interactions With CD4+ T Cells Promote the Development of Functional Osteoclasts From Murine CD11c+ Dendritic Cells. J Immunol (2006) 177(5):3314–26. 10.4049/jimmunol.177.5.3314 16920972

[120] Ono T Takayanagi H. Osteoimmunology in Bone Fracture Healing. Curr Osteoporosis Rep (2017) 15(4):367–75. 10.1007/s11914-017-0381-0 28647888

[121] Jin Y Wi HJ Choi M-H Hong S-T Bae YM. Regulation of Anti-Inflammatory Cytokines IL-10 and TGF-β in Mouse Dendritic Cells Through Treatment With Clonorchis Sinensis Crude Antigen. Exp Mol Med (2014) 46(1):e74–e. 10.1038/emm.2013.144 PMC3909892 24480801

[122] Dumitriu IE Dunbar DR Howie SE Sethi T Gregory CD. Human Dendritic Cells Produce TGF-β1 Under the Influence of Lung Carcinoma Cells and Prime the Differentiation of CD4+CD25+Foxp3+ Regulatory T Cells. J Immunol (2009) 182(5):2795–807. 10.4049/jimmunol.0712671 19234174

[123] Hughes DE Dai A Tiffee JC Li HH Mundy GR Boyce BF. Estrogen Promotes Apoptosis of Murine Osteoclasts Mediated by TGF–β. Nat Med (1996) 2(10):1132–6. 10.1038/nm1096-1132 8837613

[124] Nathan C. Neutrophils and Immunity: Challenges and Opportunities. Nat Rev Immunol (2006) 6(3):173–82. 10.1038/nri1785 16498448

[125] Selders GS Fetz AE Radic MZ Bowlin GL. An Overview of the Role of Neutrophils in Innate Immunity, Inflammation and Host-Biomaterial Integration. Regenerative Biomaterials (2017) 4(1):55–68. 10.1093/rb/rbw041 28149530 PMC5274707

[126] Wagner C Iking-Konert C Denefleh B Stegmaier S Hug F Hansch GM. Granzyme B and Perforin: Constitutive Expression in Human Polymorphonuclear Neutrophils. Blood (2004) 103(3):1099–104. 10.1182/blood-2003-04-1069 14512315

[127] Kaplan MJ Radic M. Neutrophil Extracellular Traps: Double-Edged Swords of Innate Immunity. J Immunol (2012) 189(6):2689–95. 10.4049/jimmunol.1201719 PMC3439169 22956760

[128] Nauseef WM Borregaard N. Neutrophils at Work. Nat Immunol (2014) 15(7):602–11. 10.1038/ni.2921 24940954

[129] Scapini P Cassatella MA. Social Networking of Human Neutrophils Within the Immune System. Blood (2014) 124(5):710–9. 10.1182/blood-2014-03-453217 24923297

[130] Mantovani A Cassatella MA Costantini C Jaillon S. Neutrophils in the Activation and Regulation of Innate and Adaptive Immunity. Nat Rev Immunol (2011) 11(8):519–31. 10.1038/nri3024 21785456

[131] Hajishengallis G Moutsopoulos NM Hajishengallis E Chavakis T. Immune and Regulatory Functions of Neutrophils in Inflammatory Bone Loss. Semin Immunol (2016) 28(2):146–58. 10.1016/j.smim.2016.02.002 PMC4867283 26936034

[132] Poubelle PE Chakravarti A Fernandes MJ Doiron K Marceau AA. Differential Expression of RANK, RANK-L, and Osteoprotegerin by Synovial Fluid Neutrophils From Patients With Rheumatoid Arthritis and by Healthy Human Blood Neutrophils. Arthritis Res Ther (2007) 9(2):R25. 10.1186/ar2137 17341304 PMC1906801

[133] Iking-Konert C Ostendorf B Sander O Jost M Wagner C Joosten L. Transdifferentiation of Polymorphonuclear Neutrophils to Dendritic-Like Cells at the Site of Inflammation in Rheumatoid Arthritis: Evidence for Activation by T Cells. Ann Rheum Dis (2005) 64(10):1436–42. 10.1136/ard.2004.034132 PMC1755243 15778239

[134] Moutsopoulos NM Konkel J Sarmadi M Eskan MA Wild T Dutzan N. Defective Neutrophil Recruitment in Leukocyte Adhesion Deficiency Type I Disease Causes Local IL-17-Driven Inflammatory Bone Loss. Sci Trans Med (2014) 6(229):229ra40. 10.1126/scitranslmed.3007696 PMC4090608 24670684

[135] Geissler S Textor M Stumpp S Seitz S Lekaj A Brunk S. Loss of Murine Gfi1 Causes Neutropenia and Induces Osteoporosis Depending on the Pathogen Load and Systemic Inflammation. PloS One (2018) 13(6):e0198510. 10.1371/journal.pone.0198510 29879182 PMC5991660

[136] Fulkerson PC Rothenberg ME. Targeting Eosinophils in Allergy, Inflammation and Beyond. Nat Rev Drug Discov (2013) 12(2):117–29. 10.1038/nrd3838 PMC3822762 23334207

[137] Arshi S Ghalehbaghi B Kamrava S-K Aminlou M. Vitamin D Serum Levels in Allergic Rhinitis: Any Difference From Normal Population? Asia Pacif Allergy (2012) 2(1):45–8. 10.5415/apallergy.2012.2.1.45 PMC3269601 22348206

[138] Azimi A Ghajarzadeh M Sahraian MA Mohammadifar M Roostaei B Samani SMV. Effects of Vitamin D Supplements on IL-10 and INFgamma Levels in Patients With Multiple Sclerosis: A Systematic Review and Meta-Analysis. Maedica (2019) 14(4):413–7. 10.26574/maedica.2019.14.4.413 PMC7035439 32153675

[139] Rossetti D Isoldi S Oliva S. Eosinophilic Esophagitis: Update on Diagnosis and Treatment in Pediatric Patients. Pediatr Drugs (2020) 22(4):343–56. 10.1007/s40272-020-00398-z 32519266

[140] Saha S Brightling CE. Eosinophilic Airway Inflammation in COPD. Int J Chronic Obstructive Pulmonary Dis (2006) 1(1):39–47. 10.2147/copd.2006.1.1.39 PMC2706606 18046901

[141] Loke YK Cavallazzi R Singh S. Risk of Fractures With Inhaled Corticosteroids in COPD: Systematic Review and Meta-Analysis of Randomised Controlled Trials and Observational Studies. Thorax (2011) 66(8):699–708. 10.1136/thx.2011.160028 21602540

[142] Otaki F Daniel W Geno DM Tholen C Alexander JA. Interval Bone Density in Patients With Eosinophilic Esophagitis on Steroids: 368. Off J Am Coll Gastroenterol | ACG (2017) 112:S197. 10.14309/00000434-201710001-00368

[143] Nagata M Nakagome K Soma T. Mechanisms of Eosinophilic Inflammation. Asia Pacif Allergy (2020) 10(2):e14. 10.5415/apallergy.2020.10.e14 PMC7203432 32411579

[144] Amber KT Valdebran M Kridin K Grando SA. The Role of Eosinophils in Bullous Pemphigoid: A Developing Model of Eosinophil Pathogenicity in Mucocutaneous Disease. Front Med (2018) 5:201. 10.3389/fmed.2018.00201 PMC6048777 30042946

[145] Murdaca G Greco M Tonacci A Negrini S Borro M Puppo F. IL-33/IL-31 Axis in Immune-Mediated and Allergic Diseases. Int J Mol Sci (2019) 20(23):5856. 10.3390/ijms20235856 PMC6929191 31766607

[146] Krystel-Whittemore M Dileepan KN Wood JG. Mast Cell: A Multi-Functional Master Cell. Front Immunol (2015) 6:620. 10.3389/fimmu.2015.00620 26779180 PMC4701915

[147] Galli SJ Tsai M. IgE and Mast Cells in Allergic Disease. Nat Med (2012) 18(5):693–704. 10.1038/nm.2755 22561833 PMC3597223

[148] Kroner J Kovtun A Kemmler J Messmann JJ Strauss G Seitz S. Mast Cells Are Critical Regulators of Bone Fracture-Induced Inflammation and Osteoclast Formation and Activity. J Bone Mineral Research: Off J Am Soc Bone Mineral Res (2017) 32(12):2431–44. 10.1002/jbmr.3234 28777474

[149] Fallon MD Whyte MP Craig RB Jr Teitelbaum SL. Mast-Cell Proliferation in Postmenopausal Osteoporosis. Calcified Tissue Int (1983) 35(1):29–31. 10.1007/BF02405002 6839188

[150] McKenna MJ. Histomorphometric Study of Mast Cells in Normal Bone, Osteoporosis and Mastocytosis Using a New Stain. Calcified Tissue Int (1994) 55(4):257–9. 10.1007/BF00310402 7529657

[151] Lesclous P Guez D Llorens A Saffar JL. Time-Course of Mast Cell Accumulation in Rat Bone Marrow After Ovariectomy. Calcified Tissue Int (2001) 68(5):297–303. 10.1007/BF02390837 11683537

[152] Lesclous P Saffar JL. Mast Cells Accumulate in Rat Bone Marrow After Ovariectomy. Cells Tissues Organs (1999) 164(1):23–9. 10.1159/000016639 10940670

[153] Tyan ML. Effect of Promethazine on Lumbar Vertebral Bone Mass in Postmenopausal Women. J Internal Med (1993) 234(2):143–8. 10.1111/j.1365-2796.1993.tb00723.x 8340736

[154] Zaitsu M Narita S Lambert KC Grady JJ Estes DM Curran EM. Estradiol Activates Mast Cells *via* a Non-Genomic Estrogen Receptor-Alpha and Calcium Influx. Mol Immunol (2007) 44(8):1977–85. 10.1016/j.molimm.2006.09.030 PMC2603032 17084457

[155] Rivellese F Nerviani A Rossi FW Marone G Matucci-Cerinic M de Paulis A. Mast Cells in Rheumatoid Arthritis: Friends or Foes? Autoimmun Rev (2017) 16(6):557–63. 10.1016/j.autrev.2017.04.001 28411167

[156] Feyerabend TB Weiser A Tietz A Stassen M Harris N Kopf M. Cre-Mediated Cell Ablation Contests Mast Cell Contribution in Models of Antibody- and T Cell-Mediated Autoimmunity. Immunity (2011) 35(5):832–44. 10.1016/j.immuni.2011.09.015 22101159

[157] de Lange-Brokaar BJ Kloppenburg M Andersen SN Dorjee AL Yusuf E Herb-van Toorn L. Characterization of Synovial Mast Cells in Knee Osteoarthritis: Association With Clinical Parameters. Osteoarthritis Cartilage (2016) 24(4):664–71. 10.1016/j.joca.2015.11.011 26671522

[158] Wang Q Lepus CM Raghu H Reber LL Tsai MM Wong HH. IgE-Mediated Mast Cell Activation Promotes Inflammation and Cartilage Destruction in Osteoarthritis. eLife (2019) 8:1–23. 10.7554/eLife.39905 PMC6516833 31084709

[159] Nakano S Mishiro T Takahara S Yokoi H Hamada D Yukata K. Distinct Expression of Mast Cell Tryptase and Protease Activated Receptor-2 in Synovia of Rheumatoid Arthritis and Osteoarthritis. Clin Rheumatol (2007) 26(8):1284–92. 10.1007/s10067-006-0495-8 17205215

[160] Lee H Kashiwakura J Matsuda A Watanabe Y Sakamoto-Sasaki T Matsumoto K. Activation of Human Synovial Mast Cells From Rheumatoid Arthritis or Osteoarthritis Patients in Response to Aggregated IgG Through Fcgamma Receptor I and Fcgamma Receptor II. Arthritis Rheum (2013) 65(1):109–19. 10.1002/art.37741 23055095

[161] Lindholm R Lindholm S Liukko P. Fracture Healing and Mast Cells. I. The Periosteal Callus in Rats. Acta Orthopaedica Scand (1967) 38(2):115–22. 10.3109/17453676708989624 4166390

[162] Banovac K Renfree K Makowski AL Latta LL Altman RD. Fracture Healing and Mast Cells. J Orthopaedic Trauma (1995) 9(6):482–90. 10.1097/00005131-199509060-00005 8592261

[163] Scoville SD Freud AG Caligiuri MA. Modeling Human Natural Killer Cell Development in the Era of Innate Lymphoid Cells. Front Immunol (2017) 8:360. 10.3389/fimmu.2017.00360 28396671 PMC5366880

[164] Brandstadter JD Yang Y. Natural Killer Cell Responses to Viral Infection. J Innate Immun (2011) 3(3):274–9. 10.1159/000324176 PMC3128146 21411975

[165] Trinchieri G. Biology of Natural Killer Cells. Adv Immunol (1989) 47:187–376. 10.1016/S0065-2776(08)60664-1 2683611 PMC7131425

[166] Parham P. MHC Class I Molecules and KIRs in Human History, Health and Survival. Nat Rev Immunol (2005) 5(3):201–14. 10.1038/nri1570 15719024

[167] Fauriat C Long EO Ljunggren HG Bryceson YT. Regulation of Human NK-Cell Cytokine and Chemokine Production by Target Cell Recognition. Blood (2010) 115(11):2167–76. 10.1182/blood-2009-08-238469 PMC2844017 19965656

[168] de Matos CT Berg L Michaelsson J Fellander-Tsai L Karre K Soderstrom K. Activating and Inhibitory Receptors on Synovial Fluid Natural Killer Cells of Arthritis Patients: Role of CD94/NKG2A in Control of Cytokine Secretion. Immunology (2007) 122(2):291–301. 10.1111/j.1365-2567.2007.02638.x 17521371 PMC2266001

[169] Tak PP Kummer JA Hack CE Daha MR Smeets TJ Erkelens GW. Granzyme-Positive Cytotoxic Cells are Specifically Increased in Early Rheumatoid Synovial Tissue. Arthritis Rheum (1994) 37(12):1735–43. 10.1002/art.1780371205 7986219

[170] McInnes IB Leung BP Sturrock RD Field M Liew FY. Interleukin-15 Mediates T Cell-Dependent Regulation of Tumor Necrosis Factor-Alpha Production in Rheumatoid Arthritis. Nat Med (1997) 3(2):189–95. 10.1038/nm0297-189 9018238

[171] Feng S Madsen SH Viller NN Neutzsky-Wulff AV Geisler C Karlsson L. Interleukin-15-Activated Natural Killer Cells Kill Autologous Osteoclasts *via* LFA-1, DNAM-1 and TRAIL, and Inhibit Osteoclast-Mediated Bone Erosion *In Vitro* . Immunology (2015) 145(3):367–79. 10.1111/imm.12449 PMC4479536 25684021

[172] Kurachi T Morita I Murota S. Involvement of Adhesion Molecules LFA-1 and ICAM-1 in Osteoclast Development. Biochim Biophys Acta (1993) 1178(3):259–66. 10.1016/0167-4889(93)90202-Z 7779165

[173] Colonna M. Innate Lymphoid Cells: Diversity, Plasticity, and Unique Functions in Immunity. Immunity (2018) 48(6):1104–17. 10.1016/j.immuni.2018.05.013 PMC6344351 29924976

[174] Mjosberg J Spits H. Human Innate Lymphoid Cells. J Allergy Clin Immunol (2016) 138(5):1265–76. 10.1016/j.jaci.2016.09.009 27677386

[175] Monticelli LA Sonnenberg GF Abt MC Alenghat T Ziegler CG Doering TA. Innate Lymphoid Cells Promote Lung-Tissue Homeostasis After Infection With Influenza Virus. Nat Immunol (2011) 12(11):1045–54. 10.1038/ni.2131 PMC3320042 21946417

[176] Turner JE Morrison PJ Wilhelm C Wilson M Ahlfors H Renauld JC. IL-9-Mediated Survival of Type 2 Innate Lymphoid Cells Promotes Damage Control in Helminth-Induced Lung Inflammation. J Exp Med (2013) 210(13):2951–65. 10.1084/jem.20130071 PMC3865473 24249111

[177] Stark MA Huo Y Burcin TL Morris MA Olson TS Ley K. Phagocytosis of Apoptotic Neutrophils Regulates Granulopoiesis *via* IL-23 and IL-17. Immunity (2005) 22(3):285–94. 10.1016/j.immuni.2005.01.011 15780986

[178] Zheng Y Valdez PA Danilenko DM Hu Y Sa SM Gong Q. Interleukin-22 Mediates Early Host Defense Against Attaching and Effacing Bacterial Pathogens. Nat Med (2008) 14(3):282–9. 10.1038/nm1720 18264109

[179] Kuwabara T Ishikawa F Kondo M Kakiuchi T. The Role of IL-17 and Related Cytokines in Inflammatory Autoimmune Diseases. Mediators Inflamm (2017) 2017:3908061. 10.1155/2017/3908061 28316374 PMC5337858

[180] Fang W Zhang Y Chen Z. Innate Lymphoid Cells in Inflammatory Arthritis. Arthritis Res Ther (2020) 22(1):25. 10.1186/s13075-020-2115-4 32051038 PMC7017550

[181] Leijten EF van Kempen TS Boes M Michels-van Amelsfort JM Hijnen D Hartgring SA. Brief Report: Enrichment of Activated Group 3 Innate Lymphoid Cells in Psoriatic Arthritis Synovial Fluid. Arthritis Rheumatol (2015) 67(10):2673–8. 10.1002/art.39261 26137857

[182] Omata Y Frech M Primbs T Lucas S Andreev D Scholtysek C. Group 2 Innate Lymphoid Cells Attenuate Inflammatory Arthritis and Protect From Bone Destruction in Mice. Cell Rep (2018) 24(1):169–80. 10.1016/j.celrep.2018.06.005 29972778

[183] Omata Y Frech M Lucas S Primbs T Knipfer L Wirtz S. Type 2 Innate Lymphoid Cells Inhibit the Differentiation of Osteoclasts and Protect From Ovariectomy-Induced Bone Loss. Bone (2020) 136:115335. 10.1016/j.bone.2020.115335 32240850

[184] Wang S Xia P Chen Y Qu Y Xiong Z Ye B. Regulatory Innate Lymphoid Cells Control Innate Intestinal Inflammation. Cell (2017) 171(1):201–16.e18. 10.1016/j.cell.2017.07.027 28844693

[185] Zhang Q Chen B Yan F Guo J Zhu X Ma S. Interleukin-10 Inhibits Bone Resorption: A Potential Therapeutic Strategy in Periodontitis and Other Bone Loss Diseases. BioMed Res Int (2014) 2014:284836. 10.1155/2014/284836 24696846 PMC3947664

[186] Kasagi S Chen W. TGF-Beta1 on Osteoimmunology and the Bone Component Cells. Cell Biosci (2013) 3(1):4. 10.1186/2045-3701-3-4 23321200 PMC3565958

[187] Di Munno O Ferro F. The Effect of Biologic Agents on Bone Homeostasis in Chronic Inflammatory Rheumatic Diseases. Clin Exp Rheumatol (2019) 37(3):502–7. 30557124

[188] Kaji H. Effects of Myokines on Bone. BoneKEy Rep (2016) 5:826. 10.1038/bonekey.2016.48 27579164 PMC4954587

[189] Pathak JL Bakker AD Verschueren P Lems WF Luyten FP Klein-Nulend J. CXCL8 and CCL20 Enhance Osteoclastogenesis *via* Modulation of Cytokine Production by Human Primary Osteoblasts. PloS One (2015) 10(6):e0131041. 10.1371/journal.pone.0131041 26103626 PMC4477884

[190] Fu SC Wang P Qi MX Peng JP Lin XQ Zhang CY. The Associations of TNF-Alpha Gene Polymorphisms With Bone Mineral Density and Risk of Osteoporosis: A Meta-Analysis. Int J Rheum Dis (2019) 22(9):1619–29. 10.1111/1756-185X.13647 31273943

[191] Kotrych D Dziedziejko V Safranow K Sroczynski T Staniszewska M Juzyszyn Z. TNF-Alpha and IL10 Gene Polymorphisms in Women With Postmenopausal Osteoporosis. Eur J Obstet Gynecol Reprod Biol (2016) 199:92–5. 10.1016/j.ejogrb.2016.01.037 26914399

[192] Weitzmann MN. Bone and the Immune System. ToxicoL Pathol (2017) 45(7):911–24. 10.1177/0192623317735316 PMC5749254 29046115

[193] Zha L He L Liang Y Qin H Yu B Chang L. TNF-Alpha Contributes to Postmenopausal Osteoporosis by Synergistically Promoting RANKL-Induced Osteoclast Formation. Biomedicine Pharmacother = Biomedecine Pharmacother (2018) 102:369–74. 10.1016/j.biopha.2018.03.080 29571022

[194] O’Brien W Fissel BM Maeda Y Yan J Ge X Gravallese EM. RANK-Independent Osteoclast Formation and Bone Erosion in Inflammatory Arthritis. Arthritis Rheumatol (2016) 68(12):2889–900. 10.1002/art.39837 PMC5125876 27563728

[195] Li S Yin Y Yao L Lin Z Sun S Zhang J. TNFalpha Treatment Increases DKK1 Protein Levels in Primary Osteoblasts *via* Upregulation of DKK1 mRNA Levels and Downregulation of Mir3355p. Mol Med Rep (2020) 22(2):1017–25. 10.3892/mmr.2020.11152 PMC7339467 32468044

[196] Callaway DA Jiang JX. Reactive Oxygen Species and Oxidative Stress in Osteoclastogenesis, Skeletal Aging and Bone Diseases. J Bone Mineral Metab (2015) 33(4):359–70. 10.1007/s00774-015-0656-4 25804315

[197] Manolagas SC. From Estrogen-Centric to Aging and Oxidative Stress: A Revised Perspective of the Pathogenesis of Osteoporosis. Endocrine Rev (2010) 31(3):266–300. 10.1210/er.2009-0024 20051526 PMC3365845

[198] Locantore P Del Gatto V Gelli S Paragliola RM Pontecorvi A. The Interplay Between Immune System and Microbiota in Osteoporosis. Mediators Inflamm (2020) 2020:3686749. 10.1155/2020/3686749 32184701 PMC7061131

[199] Pacifici R. Bone Remodeling and the Microbiome. Cold Spring Harbor Perspect Med (2018) 8(4):1–20. 10.1101/cshperspect.a031203 PMC5880157 28847904

